# The Core and Accessory Genomes of *Burkholderia pseudomallei*: Implications for Human Melioidosis

**DOI:** 10.1371/journal.ppat.1000178

**Published:** 2008-10-17

**Authors:** Siew Hoon Sim, Yiting Yu, Chi Ho Lin, R. Krishna M. Karuturi, Vanaporn Wuthiekanun, Apichai Tuanyok, Hui Hoon Chua, Catherine Ong, Sivalingam Suppiah Paramalingam, Gladys Tan, Lynn Tang, Gary Lau, Eng Eong Ooi, Donald Woods, Edward Feil, Sharon J. Peacock, Patrick Tan

**Affiliations:** 1 Defense Medical and Environmental Research Institute, DSO National Laboratories, Singapore, Republic of Singapore; 2 Genome Institute of Singapore, Singapore, Republic of Singapore; 3 Mahidol-Oxford Tropical Medicine Research Unit, Faculty of Tropical Medicine, Mahidol University, Bangkok, Thailand; 4 Faculty of Medicine, University of Calgary Health Sciences Centre, Calgary, Alberta, Canada; 5 Department of Biology and Biochemistry, University of Bath, Claverton Down, Bath, United Kingdom; 6 Center for Clinical Vaccinology and Tropical Medicine, Nuffield Department of Clinical Medicine, University of Oxford, Headington, Oxford, United Kingdom; 7 Duke-NUS Graduate Medical School Singapore, Singapore, Republic of Singapore; University College Cork, Ireland

## Abstract

Natural isolates of *Burkholderia pseudomallei* (Bp), the causative agent of melioidosis, can exhibit significant ecological flexibility that is likely reflective of a dynamic genome. Using whole-genome Bp microarrays, we examined patterns of gene presence and absence across 94 South East Asian strains isolated from a variety of clinical, environmental, or animal sources. 86% of the Bp K96243 reference genome was common to all the strains representing the Bp “core genome”, comprising genes largely involved in essential functions (eg amino acid metabolism, protein translation). In contrast, 14% of the K96243 genome was variably present across the isolates. This Bp accessory genome encompassed multiple genomic islands (GIs), paralogous genes, and insertions/deletions, including three distinct lipopolysaccharide (LPS)-related gene clusters. Strikingly, strains recovered from cases of human melioidosis clustered on a tree based on accessory gene content, and were significantly more likely to harbor certain GIs compared to animal and environmental isolates. Consistent with the inference that the GIs may contribute to pathogenesis, experimental mutation of *BPSS2053*, a GI gene, reduced microbial adherence to human epithelial cells. Our results suggest that the Bp accessory genome is likely to play an important role in microbial adaptation and virulence.

## Author Summary

Melioidosis is a serious infectious disease of humans caused by *Burkholderia pseudomallei*, a soil bacterium endemic to many areas in South East Asia. Besides humans, *B. pseudomallei* is also capable of infecting many other species and can be isolated from diverse environmental sources including soil, water, and air. In this study, we used DNA microarrays to probe the stability of the *B. pseudomallei* genome in a large panel of clinical, animal, and environmental strains. We found that evidence of a highly dynamic *B. pseudomallei* genome, with up to 14% being variably present across different strains. Surprisingly, strains recovered from human patients were significantly associated with the presence of “genomic islands”, corresponding to regions of DNA directly acquired from other microorganisms. Genes on these genomic islands may thus play an important role in the pathogenesis of human melioidosis.

## Introduction

Melioidosis is a potentially fatal infectious disease of humans and animals caused by the Gram-negative bacterium *Burkholderia pseudomallei* (Bp) [1]. An environmental saphrophyte found in South East Asia, Bp infections in endemic areas may be responsible for up to 20% of deaths due to septicemia [2],[3], and Bp has been designated a Category B biothreat agent [4]. A wide spectrum of disease symptoms are associated with melioidosis often leading to late diagnosis and treatment [5]. Commonly presenting as an acute septicemic illness, chronic Bp infection is also well recognized which can be confused with TB or malignancy [6]. Besides humans, Bp has a broad host range and can infect nematodes, amoebae, dolphins, birds, swine, sheep, and gorillas [7]–[11]. Bp can also be isolated from diverse environmental sources such as soil, water, and air [12]–[17]. Identifying the molecular factors responsible for this tremendous ecologic flexibility may improve our understanding of microbial survival and adaptation, and suggest novel diagnostic and treatment strategies for melioidosis.

The phenotypic versatility of Bp is likely to be underpinned by the presence of a highly dynamic genome. For example, lateral gene transfer events may cause large-scale variations in genome content [18]. The portion of the genome that is variably present between individual strains is often termed the “accessory genome”, to distinguish these genes from genes common to all strains in a population and involved in essential functions (the “core” genome). In several microbial species, accessory genes have been shown to play key roles in host adaptation and, in the case of Bp, the accessory genome may contribute to virulence and antibiotic resistance [19]. Interestingly, previous studies indicate that in Bp, gene loss, as well as gene acquisition events, can both cause phenotypic shifts towards virulence. For example, comparisons between Bp and *B. thailandensis*, an avirulent closely related species, have shown that an important evolutionary step in the development of Bp pathogenicity was the loss of an anti-virulence arabinose assimilation cluster [20],[21]. Such findings thus raise a compelling need to accurately define the core and accessory genomes of Bp.

In other γ proteobacteria genera (*E. coli*, *Pseudomonas*, *Vibrio*), the accessory genome can encompass up to 20% of all genomic content, and similar percentages may also hold for *Burkholderia spp.* [22]–[25]. However, to date, comprehensive qualitative and quantitative studies of the core and accessory genome in Bp have not been carried out, and the full extent to which gene content differences contribute to virulence in Bp is still unclear. While some previous studies have attempted to explore these issues, they have not incorporated data from the whole genome [19], [26]–[28], or have used only a very small sample of strains [29],[30]. In this study, we performed a detailed array-based comparative genomic hybridization (aCGH) analysis of close to 100 clinical, animal and environmental Bp isolates from South East Asia. To our knowledge, this is the first time a whole genome comparative study has been applied to such a large Bp strain cohort. We found that 86% of the reference Bp K96243 genome was present in all the strains, while the remaining 14% was variably present across the strain panel. Surprisingly, isolates associated with human melioidosis exhibited a tendency to harbor certain GIs compared to isolates from either animal or environmental sources, suggesting that genes on these mobile elements might facilitate colonization of the human host. Taken collectively, our results support the notion that the Bp accessory genome may play a central role in adaptation and virulence. Besides providing important evidence concerning genes likely involved in *Burkholderia* pathogenesis, this study also raises the possibility of targeting molecular diagnostics to specific Bp accessory regions for monitoring the presence of human-virulent variants in the environment.

## Results

### Genome-wide Identification of Core and Accessory Genes in Bp Isolates

Using a previously validated Bp K96243 DNA microarray [30],[31], we generated aCGH profiles for ninety-four Bp strains isolated from human patients, animals, and environmental soils in Singapore, Malaysia or Thailand (Table S1). We applied a Gaussian Mixture Model (GMM) to the aCGH data and identified 750 out of 5369 genes (14%) as being variably present across the strain panel (see Methods and Figure S1). The variability of the 750 genes was experimentally validated by several independent methods, including bioinformatic comparisons to previously-known variable genes, comparisons against publicly available genome sequences, and experimental confirmation by targeted PCR assays (Figure S2 and Table S2).

### The Bp Core Genome Encodes Essential Processes and a Common Virulence Machinery

**Figure 1:**
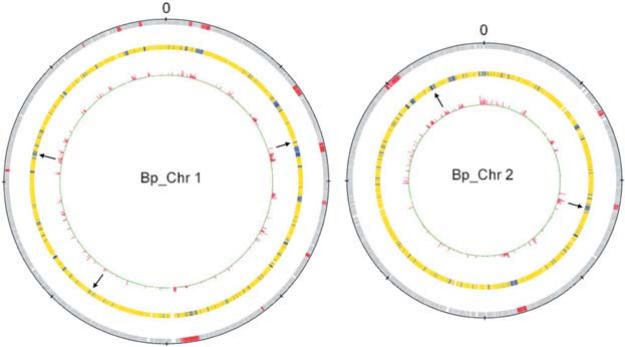
The Core and Accessory Genomes of Bp. Chromosome 1 is on the left and Chromosome 2 on the right. Both chromosomes are centered around the origin of replication. From outside to inside: Red - Computationally-identified GIs (12 on Chr 1 and 4 on Chr 2) (33); Accessory (Blue) and Core (Yellow) Genes; Internal red - False Discovery Values as assessed by GMM - A red peak indicates high variability in that genomic region (see Methods). Black arrows - Representative examples of novel indels.

86% of the Bp K96243 genes (4619) were found in all strains, representing the Bp core genome (Figure 1). Using pathway analysis, we found that the core genes were significantly over-represented in several functions necessary for basic bacterial growth and survival, including amino acid metabolism (1.52×10^−3^), inorganic ion transport (3.96×10^−3^), nucleotide metabolism (1.52×10^−2^) and protein translation (7×10^−3^) (Table 1). The core genes were also significantly enriched in genes conserved in other *Burkholderia* species (Bp, *B. mallei*, *B. thailandensis* and *B. cepacia*) (p = 8.68×10^−11^) (Text S1 and Table S3)), suggesting that a significant proportion of these Bp core genes may represent core genes in other related species as well [32]. Besides these basic housekeeping functions, the Bp core genes were also significantly enriched in commonly encountered virulence-related genes such as secretion proteins, capsular polysaccharides, exoproteins, adhesins, fimbriae and pili (p = 1.8×10^−3^) (Table 1). For example, three Bp-specific fimbrial gene clusters (*BPSL1626-1629*, *BPSL1799-1801*, *BPSS0120-0123*) were found in all strains. This finding suggests that most, if not all, Bp isolates are likely to possess a common ‘virulence machinery’. Notably, many of these conventional virulence genes are also found in other related species such as *B. thailandnesis* that although non-infectious to mammals can kill other species such as nematodes [20],[33]. This is consistent with the possibility that Bp might have descended from a pathogenic ancestor with a non-mammalian host.

**Table 1:** Enriched Functions of Core and Accessory Genes in Bp.

	Gene Distribution			
	Accessory (A)	Core (C)	Total	p-value
Total Number of Genes	750	4619	5369	
**Enriched Functions in Core Genes**				
Amino acid transport and metabolism*	37	377	414	1.5×10^−3^
Inorganic ion transport and metabolism*	16	199	215	3.96×10^−3^
Nucleotide transport and metabolism*	4	78	82	0.0152
Protein Translation*	12	158	170	0.007
Virulence Components+	30	321	351	1.83×10^−3^
**Enriched Functions in Accessory Genes**				
Paralogous Genes	73	228	301	2.25×10^−7^
Hypothetical Proteins	233	1132	1365	3.3×10^−4^

P-values were computed using a Fisher Test.

*: P-values were computed based upon the simultaneous comparison of 25 COG pathways.

**+:** Virulence genes were obtained from an annotated listing provided in Holden et al (2004) [34].

### Functional and Chromosomal Biases in the Bp Accessory Genome

14% of the Bp K96243 genome was variable across the strain panel, representing the Bp accessory genome. Since our analysis is confined to genetic elements present in the reference K96243 genome, the extent of genomic variability reported here should be regarded as a lower limit. The 750 variable genes were equally distributed between both Chromosome 1 and Chromosome 2 after normalizing for chromosome size differences. The accessory genes were significantly enriched in paralogous genes (p = 2×10^−7^) and genes encoding hypothetical proteins (p = 3×10^−4^) (Table 1). Approximately one-third (30.8%) of the accessory genes were localized to a series of previously identified “genomic islands” (GIs) in the K96243 genome [34]. GIs are regions bearing unusual sequence hallmarks, such as atypical GC content and/or dinucleotide frequencies, and are likely to have been recently acquired by lateral gene transfer. Of sixteen GIs in the K96243 genome, fourteen GIs were represented by accessory genes. In contrast, two GIs (7 and 14) were found in all strains, suggesting that GIs 7 and 14 should be regarded as part of the Bp core genome.

**Figure 2:**
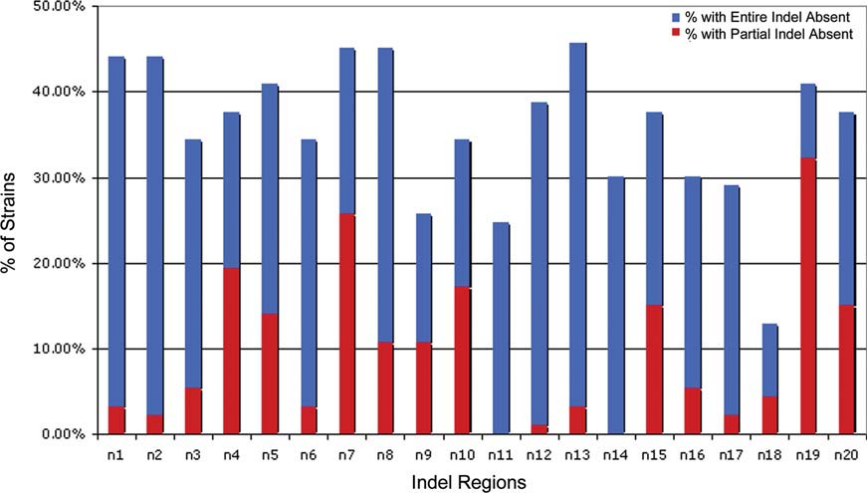
Frequency of Indels in Bp. The graph shows the percentage of strains exhibiting either a partial (red) or total (blue) absence of the indel segment (n1–n20). Blue represents the percentage of strains where the entire indel is absent. Red represents strains where the indel is only partially absent.

Besides the GIs, we also identified several novel regions of at least three contiguous probes that were absent in at least three strains. Henceforth referring to these regions as ‘indels’, we identified eight indels on chromosome 1, and twelve on chromosome 2 (Table 2). We experimentally validated two of these indels using PCR assays (Figure S3). The indels ranged in size from 1.3 to 7.5 kb, and were absent in 12.9% to 45.2% of strains (Figure 2). Three indels (n1, n4 and n11) were associated with atypical GC content (53.7–58.6%, compared to 68% for the Bp genome), and four (n2, n9, n11 and n16) carried genes characteristic of mobile genetic elements such as integrases, transposases and bacteriophage-related genes, consistent with lateral transfer. These indels may therefore share similar dynamics to the larger genomic islands, and may be considered as genomic “islets”. In other species, analogous islets which are typically <10 kb long, have been shown to play a role in virulence (e.g. the *sifA* islet in *S. typhimurium*) [35]. Of note, n16 and n18 were flanked at both their 5′and 3′ends by tandem repeat sequences, while n4, n6, n8 and n19 possessed sequence repeats at either their 5′ or 3′ ends. In some cases, the islets in the Bp genomes may actually form part of the larger GIs. For example, n2 (*BPSL0741-BPSL0744*) was located at the 5′ boundary of GI 4 (*BPSL0745-BPSL0772*), while n11 (*BPSS0395-BPSS0397*) was located immediately 3′ to GI 13 (*BPSS0378-BPSS0391A*).

**Table 2:** Novel indels in Bp.

Indel	Genes	Size (kb)	Integrase/bacteriohage/transposase	GC (%)	Presence in BT?*	Gene Functions
1	*BPSL0736 - BPSL0739*	2.7	0	**58.5**	−	Hypothetical proteins
2	*BPSL0741 - BPSL0744*	3.7	1 integrase	60.2	−	Hypothetical proteins and putative phage-related integrase
3	*BPSL1204 - BPSL1206*	2.5	0	68.2	+	Miscellaneous island; contains lipoprotein, putative amino acid transport protein and 30S ribosomal protein S15
4	*BPSL2037 - BPSL2039*	5.0	0	**53.7**	−	Hypothetical proteins
					Replaced by BTH_I2688, 2689 and 2690	
5	*BPSL2362 - BPSL2365*	4.5	0	69.6	+	Miscellaneous island; contains family U32 unassigned peptidase, putative 2-nitropropane dioxygenase, hypothetical protein and putative regulatory protein
6	*BPSL2666 - BPSL2668*	3.7	0	68.4	+	LPS biosynthesis; phogphoglucomutase, LPS biosynthesis protein and glycosyl transferase
7	*BPSL2701- BPSL2704*	4.1	0	68.3	+	Miscellaneous; contains hypothetical proteins, probable alcohol dehydrogenase and putative OmpW-family exported protein
8	*BPSL2946 - BPSL2949*	4.6	0	66.8	+	Miscellaneous; contains C4-dicarboxylate transport protein, putative GntR-family regulatory protein, cyn operon transcriptional activator (LysR-family) and carbonic anhydrase
9	*BPSS0001 - BPSS0004*	3.6	1 integrase	64.3	+	Hypothetical protein, integrase and DNA-binding protein
10	*BPSS0013 - BPSS0015*	2.4	0	68.0	+	Hypothetical proteins and glutathione S-transferase like protein
11	*BPSS0395 - BPSS0397*	1.3	2 bacteriophage proteins	**58.6**	−	Bacteriophage protein Gp49 and hypothetical protein
12	*BPSS0427 - BPSS0429*	2.7	0	66.7	+	LPS biosynthesis; contains O-acetyl transferase and glycosyl transferase (O-antigen related) and hypothetical protein
13	*BPSS0681 BPSS0683*	2.4	0	69.2	+	Miscellaneous; contains AraC family regulatory protein and hypothetical proteins
14	*BPSS0685 – BPSS0687*	4.3	0	71.3	+	Miscellaneous; contains sensor kinase protein and hypothetical protein
15	*BPSS0689 - BPSS0693*	4.1	0	69.0	+	Miscellaneous; contains MarR family regulator protein, fumarylacetoacetate (FAA) hydrolase family protein and hypothetical proteins
16	*BPSS2150 - BPSS2155*	7.5	0	69.8	+	Metabolic; contains citrate lyase, transporter proteins, zinc binding dehydrogenase and isochoristmatase.
17	*BPSS2164 - BPSS2166*	3.3	0	73.2	+	Miscellaneous; contains acylphosphatase protein and hypothetical protein
18	*BPSS2235 - BPSS2237*	3.0	0	73.5	−	Miscellaneous; contains Zinc-binding dehydrogenase and hypothetical proteins
19	*BPSS2251 - BPSS2254*	4.8	0	71.6	−	LPS biosynthesis; contains LPS biosynthesis proteins and transferases
20	*BPSS2331 - BPSS2333*	3.2	0	69.8	+	Miscellaneous; contains lipoprotein and hypothetical proteins

*: Presence indicated by +; and absence indicated by −.

Indels exhibiting atypical %GC content are indicated in bold.

Three indel regions (n6, n12 and n19) contained genes associated with LPS metabolism. Lipolysaccharides (LPS) are macromolecular components on the outer membranes of Gram-negative bacteria composed of lipid A, core oligosaccharide, and O-antigen polysaccharides [36]. LPS molecules are commonly immunogenic and have been previously implicated in virulence for numerous microbes [37],[38]. Region n6 (*BPSL2666-BPSL2668*) contains a phosphoglucomutase (*BPSL2666*), a lipopolysaccharide LPS biosynthesis protein (*BPSL2667*) and a glycosyltransferase (*BPSL2668*), and was located four genes away from a larger LPS biosynthesis cluster (*BPSL2672-BPSL2688*). Both regions n12 (*BPSS0427 - BPSS0429*) and n19 (*BPSS2245-BPSS2255*) contained two O-antigen related genes, including O-acetyltransferase and glycosyltransferase. While n12 corresponds to a previously identified type III O-PS polysaccharide gene cluster [39], the contribution of n19 genes to Bp LPS biology is currently unknown. The identification of three physically unlinked indels related to LPS metabolism provides a mechanism by which high levels of LPS diversity may be maintained in the Bp population [40].

### Unsupervised Clustering Using the Accessory Genome Distinguishes Clinical Isolates from Animal and Environmental Strains

**Figure 3:**
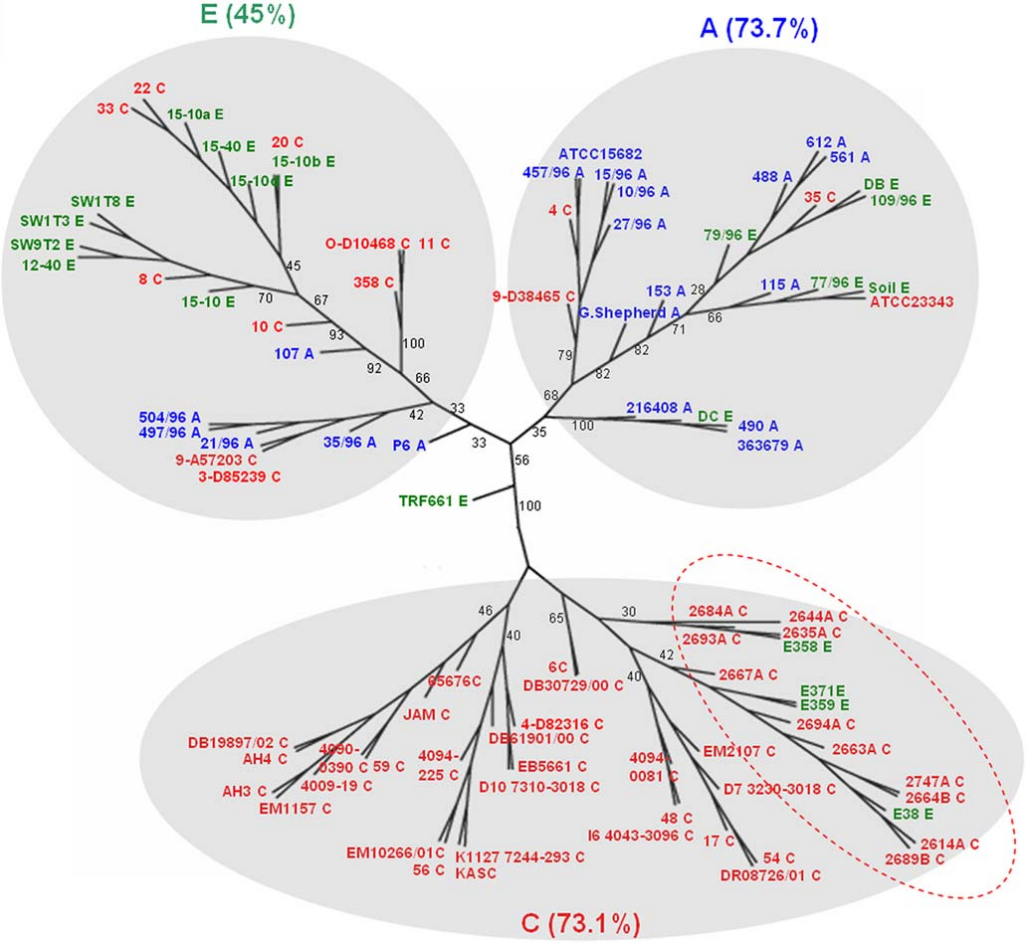
Unsupervised Accessory Genome Clustering of Bp Isolates. Clustering diagram of Bp strains on the basis of accessory genome content. The tree is contructed using MultiExperiment Viewer (MeV) version 4, based on the entire 750-gene accessory genome and combined average linkage hierarchical clustering. Clinical (labeled in red), Animal (labeled in blue) and Environmental (labeled in green) strains are indicated. Isolates from Thailand are highlighted in the red broken circle. Three broad clusters/clades are identified which are named C-clinical, A-animal, E-environmental, with the percentage of concordant strains in that cluster. Numbers on branches represent bootstrap values based on 1000 tests. The bootstrapping analysis reveals a clear distinction between the C (clinical) and A/E clusters (non-clinical - animal and environmental) (Bootstrap value = 100).

To explore if differences in accessory genome content might be associated with host adaptation or the propensity to cause disease, we applied unsupervised clustering to cluster the strains using the entire set of 750 accessory genes (“accessory genome clustering”, AGC). We identified three large AGC clusters each containing 27 to 42 strains, with each cluster containing at least 4–6 sub-branches (Figure 3). Most strikingly, the majority of human clinical isolates (73.1%) fell into one AGC cluster (Clade C), another cluster contained 73.7% of the animal isolates (Clade A), and a third cluster contained 45% of the environmental isolates (Clade E). Similar results were obtained when the clustering was repeated using either Chromosome 1 or Chromosome 2 accessory genes (Figure S4). The over-representation of human clinical isolates in the C clade was highly significant (P = 2.001×10^−14^, Fisher's exact test), and of the remaining 13 clinical isolates nine segregated within the E clade and four in the A clade. This clustering pattern is unlikely to represent differences in geographical distribution, since the majority of the clinical (65%), animal (89%) and environmental isolates (80%) were isolated in Singapore within a ∼700 km^2^ region or from nearby islands. Furthermore, clinical isolates from Thailand clustered with the other clinical isolates, despite being geographically remote. This analysis therefore suggests that strains associated with human melioidosis may possess an accessory genome distinct from most animal and environmental strains. We also note that all three clades contained environmental isolates, which is consistent with the view that the environment represents a diverse reservoir from which human and animal adapted strains emerge.

### Clinical Isolates are Associated with the Presence of Genomic Islands

**Figure 4:**
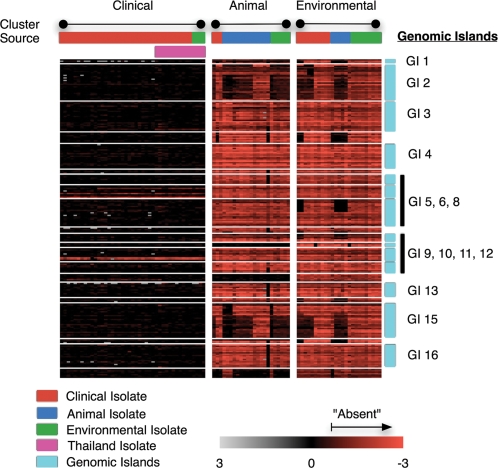
Enrichment of Genomic Islands in Clinical Isolates. Heat map representing absence and presence of GI genes in Clinical, Animal and Environmental isolates. Top row (“Cluster”): AGC clusters corresponding to clinical (left), animal (middle), and environmental (right) isolates. Second row (“Source”) Strains were color-coded according to their original source of isolation, where red = clinical, blue = animal, and green = environmental. Third row: strains highlighted in pink from Thailand. In the heat-map, black indicates gene presence and red indicates gene absence. Locations of the fourteen GIs are depicted on the right.

We then performed a supervised analysis to identify which of the 750 accessory genes were significantly different between the C and A/E clades. Of the 750 genes, 218 genes were commonly present in isolates in the C clade but absent from strains in the other two clusters (Figure 4A). Strikingly, we found that almost all of these 218 genes (85%) were localized to the GIs, with all fourteen GIs being represented. This figure (85%) is significantly higher than the 31% of all accessory genes located on GIs, raising the possibility that GIs may play an important role in determining ecological niche and host adaptation.

Is there any direct evidence that genes encoded on GIs, and which define the C clade, might play an important role in the biology or pathogenicity of Bp? Unfortunately, almost 35% of the GI genes encode ‘hypothetical’ proteins (Table S4), meaning that their function is unknown. For those genes specific to the C clade where functions could be assigned, several broad functional classes were represented. For example, GI8 contains several genes spermidine/putrescine transport genes (*potB*, *potC*, *potG*), which have been associated with biofilm formation and the regulation of Type III secretion genes [41],[42]. Type I restriction-modification enzymes are found on GI5 and GI10, and a glutathione S-transferase gene (*BPSS2048*) on GI16 may impart resistance to oxidative stress. Also supporting their potential role in Bp biology, several GI genes exhibited distinct and complex gene expression patterns during Bp growth (Text S2). However, the role of such genes in pathogenesis remains speculative. In order to explore this further, we generated an experimentally mutated strain (ATS2053) disrupted in *BPSS2053*, a GI 16 gene encoding a hemagglutinin-related protein, and determined the adherence of the mutant strain to human buccal epithelial cells. A highly significant reduction in the adherence to buccal epithelial cells was noted between the 1026b clinical isolate and the isogenic ATS2053 mutant strain (mean adherence: 1026b - 16.3±3.2 vs ATS 2053 - 4.4±1.7, p<0.001, Students t test). This finding provides evidence pointing both to the biological relevance of GI genes, but more specifically to a role of these genes in virulence.

### Comparison of Clustering Patterns Using MLST or the Accessory Genome

Finally, we examined the concordance between strain clusters defined on the basis of accessory gene content and the phylogenetic signal within the Bp core genome. We characterised 45 representative isolates by Multilocus Sequence Typing (MLST), a typing scheme that indexes variation at seven core housekeeping genes [43]. Using the previously published Bp scheme [44], we resolved the 45 isolates into 9 sequence types (ST 46, 51, 54, 84, 169, 289, 414, 422 and 423). Seven of these STs (ST51, 54, 84, 46, 169, 289, 414) have been previously observed in Malaysia, Thailand, and Singapore and two (ST422 and 423) are specific to Singapore [44],[45].

Previous analyses of MLST for Bp have highlighted the difficulties in building robust phylogenetic trees for this species, owing to a paucity of informative sites in the concatenated data and frequent homologous recombination [46]. We thus favored a categorical approach to comparing the AGC and MLST data by examining the distribution of sequence types across the three clades defined by the AGC data (Table 3). This analysis revealed that the STs are not randomly distributed between the three clusters, indicating some consistency between the MLST and AGC datasets. Most strikingly, of the 20 ST51 isolates, 17 clustered within the animal-associated clade (A), three within the clinical C clade, and none in the environmental E clade. Of the other STs where at least 4 isolates were observed, all four ST422 isolates corresponded to the C clade, and all four ST84 isolates clustered within the E clade. Finally, of the nine ST423 isolates, five clustered within the C clade and four in the E clade.

**Table 3:** Concordance of AGC Clusters and MLST Sequence Types.

		AGC Clades		
		C	A	E
**MLST Sequence Types**	ST51	3	**17**	0
	ST423	5	0	4
	ST422	**4**	0	0
	ST84	0	0	**4**
	ST169	1	0	0
	ST46	1	0	2
	ST54	0	0	1
	ST414	1	0	1
	ST289	0	0	1
	**Total**	15	17	13

Depicted are the distributions of 45 Bp strains subjected to both AGC and MLST analysis. Strain numbers in bold (eg ST51) highlight STs where the majority of strains were found in one AGC clade.

These data suggest that the animal-associated clade is likely to correspond to a single clone (ST51) and provides some evidence for concordance between STs 422 and 84 with the AGC data, although the evidence in these latter cases is equivocal due to the small number of strains. In contrast, the “split” of the ST423 isolates between the clinical and environmental clades, and the 3 ST51 isolates belonging to the clinical clade, represent clear discrepancies between the two datasets. Possible explanations for these discrepancies may represent convergence of either the MLST or the AGC data, as discussed below.

## Discussion

In this report, we present a comprehensive aCGH analysis for a large series of natural Bp isolates. We found that the accessory (variably present) portion of the Bp genome corresponds to ∼14% of the whole genome content, which is broadly similar to other γ-proteobacteria. Since this approach is limited to the detection of elements present in the Bp K96243 genome, and novel elements in query genomes are not detected, this estimated fraction of the accessory genome should be regarded as a lower bound.

In the only published study of a Bp genome sequence to date, Holden et al (2004) computationally identified 16 GIs comprising 6% of the K96243 genome [34], and our data confirm that most of these islands are indeed highly variable between strains. However, two GIs (7 and 14) were found in all strains and should thus be regarded as part of the Bp core genome. Furthermore, our data also revealed the variable presence of several other small genomic islets/indels across the two chromosomes, which might contribute to the phenotypic diversity of Bp. Notably, we observed that several indels (n6, n12 and n19) were related to LPS biology. Currently, the exact contribution of LPS to Bp virulence is unclear. For example, DeShazer et al (1998) showed that Bp type II O-PS is essential for serum resistance and virulence [47], and mice pre-immunized with Bp LPS displayed enhanced survival to a subsequent challenge [48]. In contrast, other groups have reported that Bp LPS exhibits a reduced ability to activate immune cells compared to *E. coli* LPS, suggesting that LPS might play only a minimal role in Bp virulence. It is possible that these conflicting results might reflect heterogeneity in LPS pathways resulting from the variable presence of these indels, and represent an important mechanism for host adaptation. Interestingly, while it was recently shown that type III O-PS mutants (indel n12) do not appear to exhibit significant virulence attenuation in mouse infection assays [39], we have found in preliminary work that Bp strains lacking the indel n19 LPS cluster generally exhibited lower levels of virulence compared to strains where this cluster was present (SSH, data not shown). In the AGC tree, n19 was absent both from three strains segregating as a single branch in the A clade, and from 5 strains in the C clade that segregated across multiple branches. This suggests that n19 may have been recurrently lost in different Bp lineages. Further experiments are clearly required to understand the role of these LPS clusters in Bp virulence.

We also found that the Bp strains could be clustered into distinct clades based on both the presence and absence of specific accessory genes. Of primary interest, strains belonging to the C clade of clinical isolates were largely defined by the presence of 218 genes, of which 85% are localized to the GIs. These findings provide evidence for a distinct repertoire of Bp genes that may cause a predisposition to human disease and that these genes tend to be located on GIs. Although many of the genes encoded on the GIs are of unknown function, we present experimental evidence that a strain mutated in one of these genes exhibited decreased adherence to human buccal endothelial cells, supporting a role in virulence potential. We also observed coordinated growth-associated expression of several GI genes, which is also consistent with the view that they play an important biological role. What might this biological role be? At present, we consider it most likely that this “virulent” combination of genes has likely emerged for reasons other than to cause human disease, particularly since cases of human (or animal) infection are relatively rare compared to the density of Bp in the soil. In contrast to bacteria which are obligately associated with eukaryotic hosts, soil bacteria such as Bp commonly face extreme and unpredictable biotic and abiotic challenges including extreme temperature shifts, solar radiation, variable humidity, competition for nutrients, and the requirement to survive ingestion by predatory protozoa, nematodes, the production of bacteriocides from other bacteria and phage infection. It thus seems entirely plausible that genes facilitating survival against these environmental challenges might have also indirectly enhanced the microbe's ability to colonize and “accidently” infect a human host, particularly when the host is immunocompromised [49].

Another possibility that might explain the enrichment of GIs in the clinical isolates is that Bp is undergoing cryptic cycling through normal human hosts (as opposed to the immunodeficient host), and that these GIs are selected during this host-pathogen interaction. In melioidosis-endemic NE Thailand, the majority of healthy individuals have antibodies to Bp by the age of 4 years, indicating a constant exposure to the bacterium that may occur by inoculation, inhalation or ingestion [50]. Within these normal hosts, Bp is likely to spend a period of time being exposed to the effects of the host immune response, after which the microbe may experience bacterial death, persistence, or expulsion from the host in a viable state and subsequent return to the environment. This latter process might occur through skin desquamation or urine and stool, since human excrement commonly finds its way back to the environment. Such cryptic cycling of Bp through the normal human host population could also lead to the selection of factors that promote survival *in vivo*. However, as we consider the human host to be a relatively minor component of Bp ecology, we argue that this scenario is, on balance, less likely.

The availability of both MLST and aCGH data for a representative sub-sample of isolates also provided us the opportunity to compare clade distributions defined either by accessory genome content or allelic variation in the core genome. We found that the animal associated strains largely corresponded to a single MLST clone (ST51). These isolates were assembled from three distinct sources: the Singapore zoo, the University of Malaya and a pig abbatoir in Singapore. The soil isolates corresponding to ST51 (which also clustered in the A clade) were not isolated from soil samples in proximity to the animal ST51 isolates, which suggests that this genotype is also present in the environment. The homogeneity of these isolates is therefore striking and cannot be explained simply by sampling bias. The consistency between the microarray and MLST data strongly suggest that this clade is monophyletic, and that the strains harbour similar gene repertoires by virtue of common descent.

In contrast, we also observed clear discrepancies between the MLST and aCGH clades. For example, three ST51 isolates clustered within the clinical aCGH clade, and ST423 was split between the clinical and environmental aCGH clades. There are three possibilities to explain these discrepencies: i) The MLST data represents the ancestral state which is inherited by descent into two AGC-defined clades - this is unlikely for the animal cluster as the vast majority of isolates are ST51, but might conceivably explain the ST423 split between the clinical and environmental clades. ii) Convergence of the MLST alleles - this would imply that isolates with the same ST are not identical by descent but happen to share the same combination of alleles. The presence of a few very common alleles for each gene, combined with high rates of recombination in Bp make this possibility more likely. iii) Independent convergence of gene content to one of the three clusters. Unless large numbers of genes can be transferred in single events, this possibility seems less parsimonious than (ii). More data are required to examine which of these hypotheses is most likely.

In summary, our study provides direct experimental confirmation that the Bp genome is highly plastic, and that gene acquisition and deletion are major drivers of this variability. This variability is far from random, and is functionally biased towards genes involved in mobile elements, hypothetical and paralogous genes, and LPS biosynthesis. Furthermore, genes on mobile elements may predispose individual strains, either directly or indirectly, towards causing human disease. We believe this latter result is significant in that most Bp research to date has focused on virulence components in the Bp core genome rather than genes on mobile elements. We conclude by noting that most of the Bp genome sequences currently available have been obtained from human clinical isolates. Given our results, it might be highly informative to subject a panel of animal and environmental Bp isolates to similar detailed genome analysis as well.

## Methods

### Bacterial Strains

Ninety-four Bp isolates were used in this study. These include: a) the K96243 reference strain, b) 52 clinical isolates from melioidosis patients between 1996 and 2005, c) 19 animal isolates from various species (eg monkeys, pigs, birds, and dogs) diagnosed with melioidosis between 1996 and 2000, d) 20 soil isolates from 1994 to 2003, and e) two type strains (ATCC23343 and ATCC15682). All strains were isolated in Singapore, neighboring islands, or surrounding countries (Malaysia, Thailand). The isolates were sampled from a diversity of locations and not a single site, supporting their unbiased nature (Aw Lay Tin and Joseph Tong, personal communication). Further strain information is provided in Table S1.

### Genomic DNA Extraction and Array-Based Comparative Genomic Hybridization (aCGH)

Strains were cultured on Tryptone Soy Agar (TSA) (Difco Laboratories, Detroit, Michigan) at 37°C, and genomic DNA extracted using a genomic DNA purification kit (Qiagen). The Bp DNA microarray has been previously described [29]–[31] and comprises approximately 16,000 PCR-amplified array probes representing all 5742 predicted genes in the K96243 genome printed in duplicate. Test genomic DNA (2 µg) was fluorescently labeled with Cy3-dCTP (Amersham Pharmacia Biotech) using nick-translation and co-hybridized to the array with an equal quantity of Cy5-dCTP (Amersham Pharmacia Biotech) labeled reference K96243 DNA. The absence of significant dye-bias artifacts was confirmed by analyzing reciprocal dye-swap hybridizations for 10 isolates data not shown, also see ref [29]. Raw fluorescence data was acquired using an Axon scanner with GENEPIX v4.0 software (Axon Instruments, Redwood City, CA).

### Microarray Data Preprocessing

Individual arrays were internally normalized between the Cy3 and Cy5 channels by LOWESS normalization, and the entire dataset was cross-normalized by median-scaling each array to the same Cy3/Cy5 ratio. To filter the microarray data, we eliminated probes exhibiting a missing value score across >40% of samples (indicating that they were not reliably measured), and probes whose genomic loci were redundant with other probes. This data filtering procedure generated a final high-quality data set of 5369 non-redundant probes. The entire microarray data set is available at the Gene Expression Omnibus database under accession number GSE9491.

### Identification of Accessory Genes

A Gaussian mixture model (GMM) [51] was used to identify accessory and core genes in the data set. In concept, a GMM fits a test signal distribution (such as microarray data) to either a single or double gaussian curve, and the likelihood that the distribution corresponds to a single curve is computed. The GMM was applied in two stages. First, p-values were computed using the aCGH profiles of each individual array spot, following a chi-square distribution with 3 degrees of freedom under the null hypothesis that the data distribution of the spot follows a 1-gaussian distribution. Second, since each probe was spotted twice on the array, we obtained composite p-values of each array probe using Inverse Chi-square Meta-Analysis [52], squaring the p-values of both spots belonging to the same probe. This latter statistic follows a chi-square distribution with 4 degrees of freedom. All p-values were corrected for multiple-hypothesis testing according to the Benjamini-Hocheberg procedure [53]. A cut-off of p≤1.83E-08 was selected to define the top 750 most highly variable probes, representing the accessory genome.

### Pathway Analysis of Core and Accessory genes

All protein coding sequences in the Bp K96243 genome were queried by BLASTP against the Cluster of Orthologous group (COGs) database, a public bioinformatic database that groups protein sequences on the basis of phylogenetic similarity to various cellular functions, such as protein translation, DNA replication and transcription, nuclear structure and defense mechanisms (accessible at http://www.ncbi.nlm.nih.gov/COG/new/). Matches were defined as database hits with an e-value threshold of <10^−6^. Based on the COG assignments, the K96243 proteins were assigned to functional categories. Fisher's exact tests were used to identify significantly overrepresented COG categories in either the core or accessory genes. To identify conserved genes (metagenes) across four *Burkholderia* species, we queried the 3460 Chr 1 and 2395 Chr 2 ORFs in the Bp K96243 genome against the *B. cenocepacia* (Bc), *B. mallei* (Bm), and *B. thailandensis* (Bt) genomes using tblastn [32] (Text S1). To minimize the number of ambiguous predictions including ORFs with matches to multiple genomic locations, we constrained the resulting matches to have I) a minimum length of 50 amino acids, II) a minimal e-value cut-off of 1e-6 and III) a minimum percent identity of 50%. Homology assignments returned 2675 genes and were validated by a reciprocal blast assay resulting in 2590 genes. Control analyses using either Bc, Bm or Bt as starting reference genomes yielded similar metagene sets (data not shown). Paralogous genes were identified using the CD-HIT program [54] as genes with >60% identity to one another, following established studies [55],[56]. Tandem repeat regions in the K96243 genome were identified using the Tandem Repeats Finder program [57].

### Clustering Analysis

Phylogenetic trees based on aCGH profiles were constructed using MultiExperiment Viewer (MeV) version 4 (http://www.tm4.org/mev.html) using an average linkage clustering algorithm with a Euclidean distance metric. Support trees were based on 1000 bootstrap samples. Neighbor-joining trees based on MLST sequence data were constructed by MEGA ver. 2.1 software using the Kimura-2-parameter method of distance estimation. eBURST v3 (http://eburst.mlst.net) was used to demonstrate relationships between closely related STs (those differing at only a single locus) [58],[59], with the tree files visualized using PhyloDraw [60].

### Construction of Mutants

The *BPSS2053* (*fhaB*) gene was disrupted in strain DD503, an isogenic derivative of wild-type 1026b. In DD503, the amr locus, encoding a multidrug efflux system, has been experimentally deleted [61]. The increased antibiotic susceptibility of DD503 makes it a useful strain for allelic exchange experiments as it allows the use of currently available allelic exchange vectors. There is no significant difference in virulence between the1026b parent strain and DD503 [61]. A 1036-bp internal region of the *BPSS2053* (*fhaB*) gene was amplified by PCR using primers 53F:TGGTGGTGCAAGAGAATGGC and 53R:ATCGTGACCGATTGCTTGCC from Bp 1026b chromosomal DNA as previously described [21]. The PCR product was cloned into pCR2.1-TOPO (Invitrogen Life Technologies, Burlington, Ontario, Canada) according to the manufacturer's instructions. The internal region from *BPSS2053* was cloned as an EcoR1 fragment into pGSV3-lux, a suicide vector containing a promoterless lux operon as a reporter, to create pATS2053. The recombinant plasmid pATS2053 was transformed into *E. coli* SM10λpir [62]. Transformed *E. coli* containing pATS2053 were conjugated with Bp DD503, and transconjugants selected on LB-gentamicin-polymyxin B agar. The transconjugants were screened for lux-mediated light production by assaying 100 µl of overnight broth cultures of individual colonies. One of the light-producing transconjugant strains was designated as Bp ATS2053.

### Adherence Assays

Adherence of *BPSS2053* (*fhaB*) mutants (Bp ATS2053) to human buccal epithelial cells *in vitro* were compared against wild-type parental Bp 1026b as previously described [63]. Briefly, buccal epithelial cells from healthy control individuals were isolated by vigorous scraping of the buccal mucosa with a cotton-tipped swab. The swabs were placed into phosphate buffered saline (PBS), transported to the laboratory, and the epithelial cells were incubated *in vitro* with bacteria at a ratio of 100 bacteria to 1 epithelial cell for 1 h at 37C in a shaking water bath. Unattached bacteria were removed from the mixture by repeated washing with PBS and centrifugation. Bacteria per cell were counted following staining of the bacteria-cell mixture with methylene blue by counting the number of bacteria attached to each of 50 cells and obtaining a mean number of bacteria/cell.

### Multilocus Sequence Typing (MLST)

MLST on 45 strains was performed as described in Godoy et al (2003) [44] using primer pairs for seven housekeeping genes (*ace*, *gltB*, *gmhD*, *lepA*, *lipA*, *narK ndh*) on Bp chromosome 1. A complete list of primer pair sequences and PCR conditions is provided in Table S5. Alleles at each of the MLST loci were assigned using the *B. pseudomallei* MLST website (http://bpseudomallei.mlst.net/) - each allele was assigned a different allele number and the allelic profile (string of seven integers) was used to define the sequence type (ST). Sequences that were not in the database were checked by re-sequencing, assigned as new alleles and deposited in the MLST allele database.

## Supporting Information

Figure S1 Gaussian Distribution curves of genes above and below the GMM threshold
(0.16 MB DOC)

Figure S2 Experimental and Computational Validation of Variable and Stable Genes in Bp
(0.37 MB DOC)

Figure S3 Validation of 2 novel indel regions (n5 and n7) using PCR
(0.33 MB DOC)

Figure S4 AGC Clusters Based on Chromosome 1 or Chromosome 2
(0.25 MB DOC)

Table S1 Bp Isolates Used in this Study
(0.17 MB DOC)

Table S2 Sequence identities between Bp K96243 and five Bp strains (S13, BP 1710a, 1710b, 1655, Pasteur)
(0.03 MB DOC)

Table S3 Analysis of conserved metagenes* in the set of variable genes and non-variable genes in the *B. pseudomallei* genome, in the presence and absence of the GI genes
(0.04 MB DOC)

Table S4 Genes Present in Strains Associated with the AGC Clinical Clade
(0.24 MB DOC)

Table S5 Primer pairs for amplification of housekeeping loci in multilocus sequence typing analysis (MLST)
(0.04 MB DOC)

Text S1 Supplementary Methods
(0.04 MB DOC)

Text S2 Expression Patterns of GI Genes During Bp Growth
(0.57 MB DOC)
