# Supplementary material for: The Core and Accessory Genomes of Burkholderia pseudomallei: Implications for Human Melioidosis
Source: PLoS Pathog. 2008 Oct 17;4(10):e1000178. doi: 10.1371/journal.ppat.1000178 (PMC2564834; doi:10.1371/journal.ppat.1000178)
Supplement: Figure S1 — Gaussian Distribution curves of genes above and below the GMM threshold (0.16 MB DOC) [file ppat.1000178.s001.doc]

**Figure S1 : Gaussian Distribution curves of genes above and below the GMM threshold**

We performed our analysis emphasizing a high degree of specificity, and to minimize the possibility of false-positives. Each gene was plotted with its frequency of observation (density, y-axis) against its normalized aCGH ratio (x-axis), and ranked by its likelihood of departure from a single gaussian distribution (see Methods in Main Text). A visual inspection of the distributions revealed that genes below rank 750 were largely associated with double distributions (top graphs), while genes above rank 750 generally exhibited single distributions (bottom grahs). For double distributions, an arbitrary aCGH ratio of 0 was ascribed to one of the peaks. We chose to define the top ranked 750 genes as ‘variable’, corresponding to a corrected p-value threshold of 1.83x10^-08^, and to treat genes above this threshold as ‘stable’.

Below 750 threshold (Variable)

Above 750 threshold (Stable)
