# Supplementary material for: The Core and Accessory Genomes of Burkholderia pseudomallei: Implications for Human Melioidosis
Source: PLoS Pathog. 2008 Oct 17;4(10):e1000178. doi: 10.1371/journal.ppat.1000178 (PMC2564834; doi:10.1371/journal.ppat.1000178)
Supplement: Figure S2 — Experimental and Computational Validation of Variable and Stable Genes in Bp (0.37 MB DOC) [file ppat.1000178.s002.doc]

**Figure S2 : Experimental and Computational Validation of Variable and Stable Genes in Bp**

(A) GMM distribution curves of 4 genes associated with either very low (*BPSL2707*), moderate (*BPSL0137*), high (*BPSL1113*) and very high (*BPSS2295*) p-values (p)/ranks. Genes below the p-value cut-off of ≤1.83E-08 (Rank <750) are characterized by a double Gaussian distribution curve. Beyond this cutoff, genes were primarily associated with single Gaussian distributions (B) PCR validation of GMM defined variable probes. Six genes were selected and tested for their presence and absence among six different *B. pseudomallei* isolates. Lane1 -100bp ladder; Lanes 2 to 4 -three *B. pseudomallei* isolates where the gene was predicted to be present; Lane 5 to 7 –three *B. pseudomallei* isolates which the gene was predicted to be absent; Lane 8 –K96243 (positive control); Lane 9 -dH_2_O (negative control). Four genes (BPSL2707, BPSL0137, BPSS0681, BPSS2076) were below the Rank 750 cut-off. False positives and negatives were observed for the two genes (BPSL0900, BPSS2295) above this cut-off. (C) Distribution of GI genes. The horizontal axis refers to the genes ranked by GMM from 1-5369. The vertical axis refers to the cumulative percentage of known GI genes identified at that particular rank. 64.71% of known GI genes could be re-identified within the 750 Bp-variable genes. (D) Sequence identities between Bp K96243 and five Bp strains (S13, BP 1710a, 1710b, 1655, Pasteur) of all genes and the and 750 Bp variable genes. The average sequence identities for the variable probes are significantly lower than the average sequence identities for the full genome in all five Bp strains (t-test: P<0.001).

To validate our selection of the 750-gene threshold, we employed three independent experimental and computational methods. First, we performed targeted PCR experiments on 4 predicted variable genes (*BPSL2707, BPSL0137, BPSS0681, BPSS2076*) with ranks below 750, and 2 predicted stable genes with ranks above 750 (*BPSL0900, BPSS2295*) (Figure S2A). For each gene, we designed oligonucleotide primers based on the Bp K96243 reference genome, and tested if it could be amplified in Bp strains where the gene was classified to be either variable or stable (Figure S2B). There was an excellent concordance between the microarray and PCR results for all six genes, consistent with genes below rank 750 representing truly variable genes in Bp. Second, we reasoned that the 750 Bp-variable genes should be preferentially enriched in genes located within the sixteen genomic islands (GIs) previously identified by genome analysis, given the latter’s established inter-strain variability. Indeed, the Bp-variable 750 gene set was significantly enriched in GI genes (231/357 genes, 64.7%, p=3.67x10^-180^, Chi-square) (Figure S2C). The absence of the remaining 126 GI genes in the Bp variable gene set could be due to several reasons. For example, our decision to employ a stringent threshold may have resulted in some of these GI genes being classified as false negatives. To test this, we relaxed the aCGH threshold from 750 to the top 1000 genes, but found that the GI coverage was only increased by an additional 5% from 64% to 69% (an extra 18 GI genes) (Figure S2C). This observation argues that our selected 750 gene threshold represents a reasonable balance between sensitivity and specificity. Another possibility is that despite their name, certain GIs may in reality be stably present across most Bp isolates – genes in these GIs would obviously not be considered as variable genes (see Main Text). Third, besides the K96243 reference genome, several other Bp genome sequences in various stages of completion were also available at the time of this work, providing an opportunity to evaluate the variability of the 750 gene set at the nucleotide sequence level. Using BLASTN, we compared the sequences of the 750 variable genes between K96243 and five different sequenced Bp genomes (S13, BP 1710a, 1710b, 1655, Pasteur). These five strains were not part of our original Bp South-East Asian panel, and can be considered an independent validation set. For all five strains, the average sequence identity of the 750 Bp-variable genes was significantly lower than the genome average, indicating that they are indeed hypervariable (p=1.47x10^-31^ to 6.39x10^-19^) (Figure S2D and Table S2a). This difference remained significant even after excluding genes in the previously-known GIs (P=2.46x10^-7^ to 8.49x10^-3^, t-test) (Table S2b). To determine the relative contribution of either gene retention/deletion or sequence divergence to aCGH variability, we then eliminated all K96243-specific genes from the analysis and repeated the comparisons. We found that differences between the variable set and the genome average became insignificant after removing the K96243-specific genes (data not shown), indicating that most of the variable genes detected in this study are largely due to differential genome retention and deletion events. This was further confirmed by sequence comparison of K96243 to an in-house generated whole-genome sequence of Bp strain 22 (data not shown). Taken collectively, these three independent approaches confirm the robustness of the microarray data and validate our selection threshold for the cohort of Bp-variable genes.
