# Supplementary material for: The Core and Accessory Genomes of Burkholderia pseudomallei: Implications for Human Melioidosis
Source: PLoS Pathog. 2008 Oct 17;4(10):e1000178. doi: 10.1371/journal.ppat.1000178 (PMC2564834; doi:10.1371/journal.ppat.1000178)
Supplement: Figure S3 — Validation of 2 novel indel regions (n5 and n7) using PCR (0.33 MB DOC) [file ppat.1000178.s003.doc]

**Figure S3 : Validation of 2 novel indel (nGI n5 and n7) regions using PCR.**

Each novel indel is defined as a region of at least three consecutive variable probes in at least three Bp isolates. Using independently designed PCR specific primers, each of these variable probe was confirmed for their presence and absence among six different *B. pseudomallei* isolates. Loading sequence for each gel: Lane1 -100bp ladder; Lane 2 to 4 -three *B. pseudomallei* isolates which the particular gene is present; Lane 5 to 7 –three *B. pseudomallei* isolates which the particular gene is absent; Lane 8 –K96243 (positive control); Lane 9 -dH_2_O (negative control). All 2 regions were confirmed to be variable among the six *B. pseudomallei* isolates using PCR.

**BPSL2365 (Rank263, p: 1.06E-38)**

M

153

21/96

33

54

4-D82316

K1127 7244 -293

(+)

(-)

Predicted present

Predicted absent


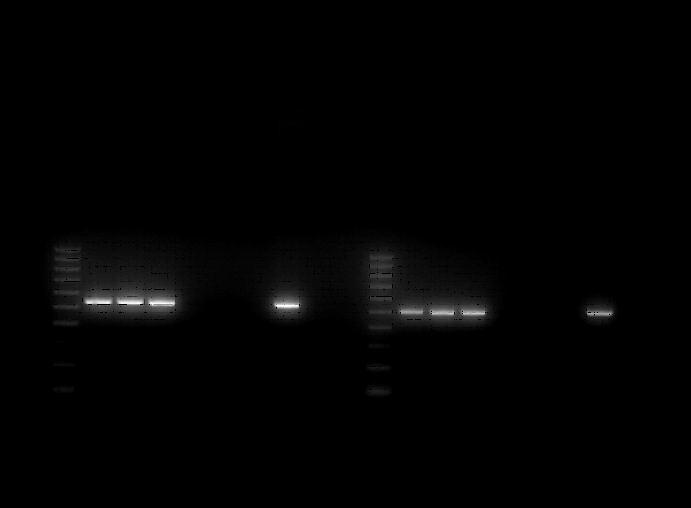


**BPSL2363 (Rank737, p: 1.31E-08)**

M

153

21/96

33

54

4-D82316

K1127 7244 -293

(+)

(-)

Predicted present

Predicted absent


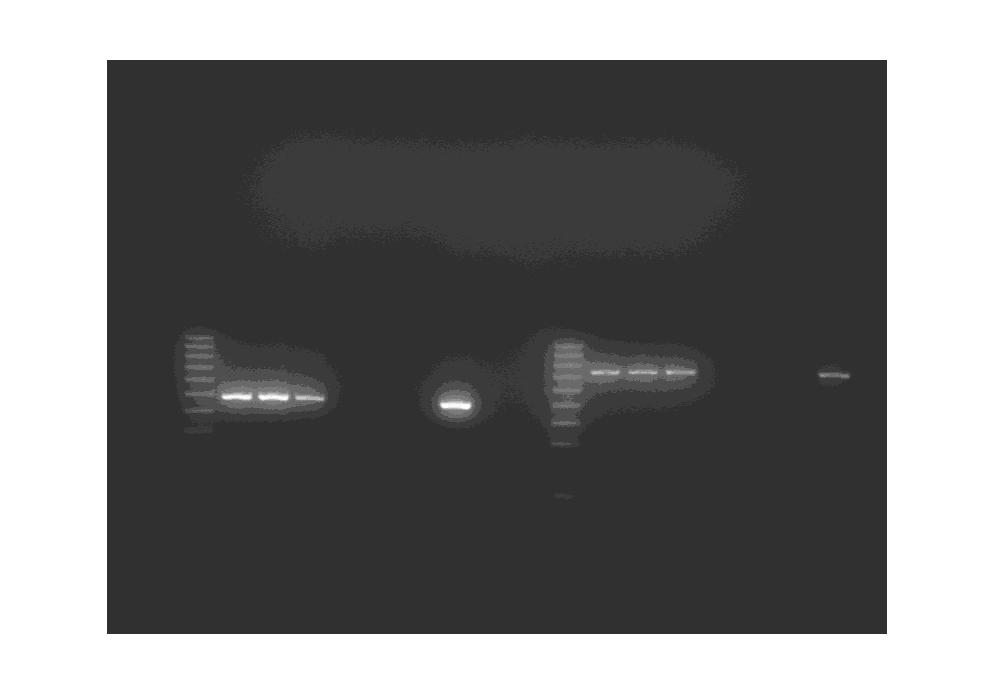


Novel

GI 5

**BPSL2364 (Rank146, p: 9.50E-53)**

M

153

21/96

33

54

4-D82316

K1127 7244 -293

(+)

(-)

Predicted present

Predicted absent


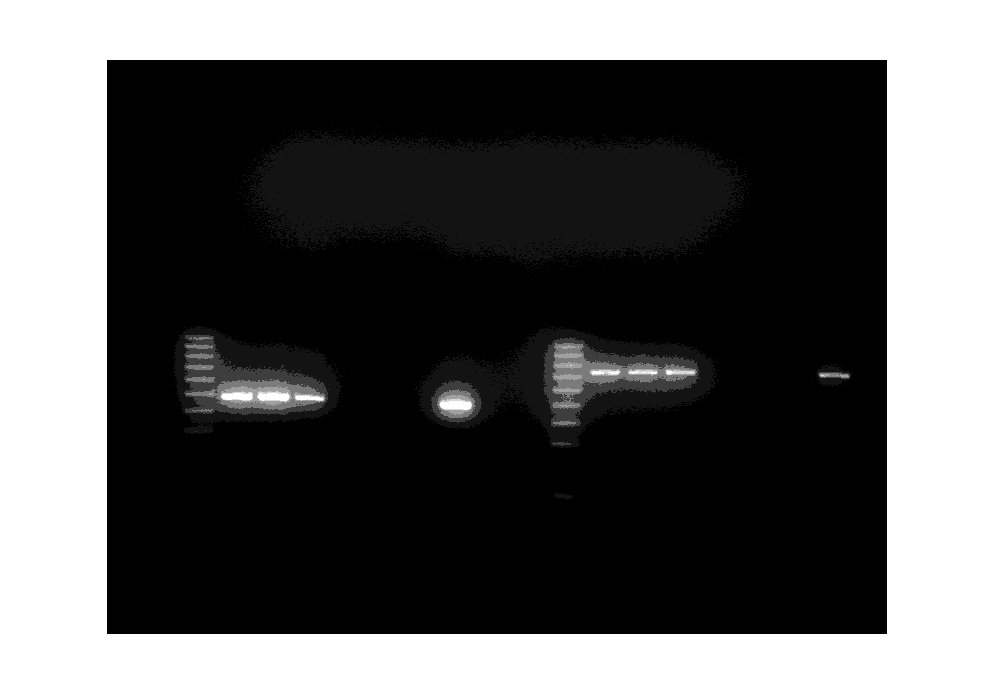


BPSL2363

BPSL2364

BPSL2365

Bp 53, 21/96, 33

BPSL23643

BPSL2364

BPSL2365

Bp 54, 4-D82316, K1127 7244-293

Novel

GI 7

**BPSL2703 (Rank272, p: 6.67E-38)**

M

21/96

9-A57203

22

DB30729/00

17

JAM

(+)

(-)

Predicted present

Predicted absent


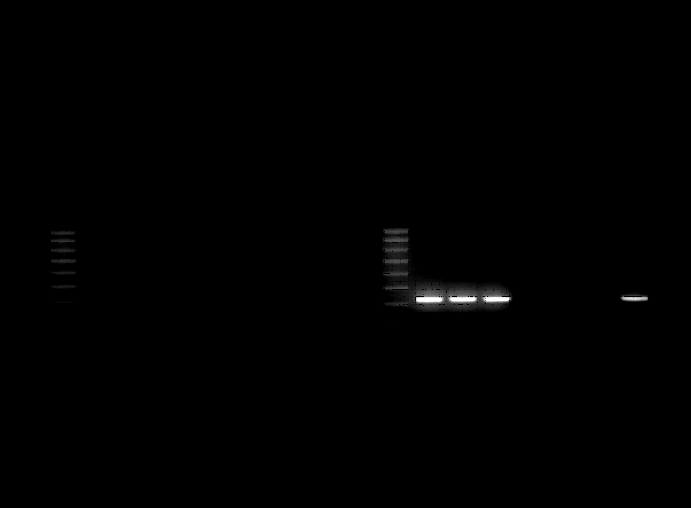


**BPSL2702 (Rank105, p: 8.35E-59)**

M

21/96

9-A57203

22

DB30729/00

17

JAM

(+)

(-)

Predicted present

Predicted absent


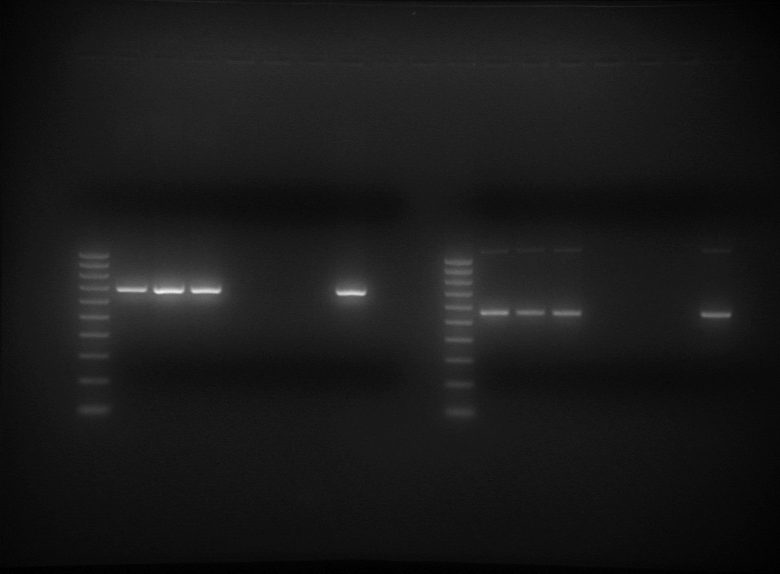


**BPSL2701 (Rank283, p: 2.38E-36)**

M

21/96

9-A57203

22

DB30729/00

17

JAM

(+)

(-)

Predicted present

Predicted absent


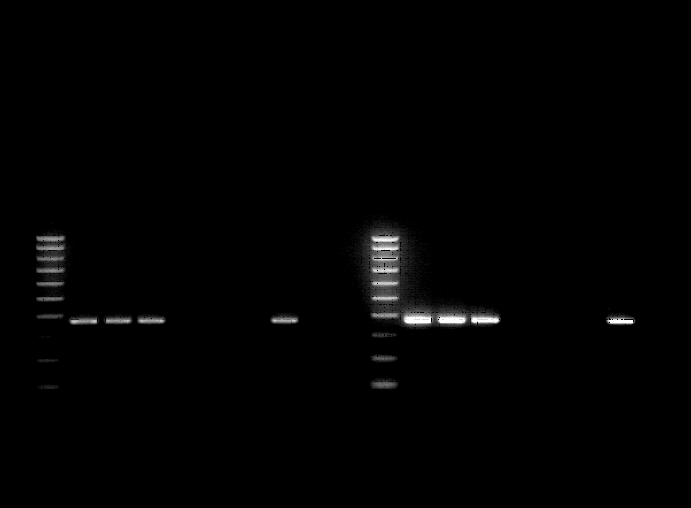


BPSL2701

BPSL2702

BPSL2703

Bp 21/96, 9-A57203, 22

BPSL2701

BPSL2702

BPSL2703

Bp DB30729/00, 17, JAM

# 
