# Supplementary material for: The Core and Accessory Genomes of Burkholderia pseudomallei: Implications for Human Melioidosis
Source: PLoS Pathog. 2008 Oct 17;4(10):e1000178. doi: 10.1371/journal.ppat.1000178 (PMC2564834; doi:10.1371/journal.ppat.1000178)
Supplement: Figure S4 — AGC Clusters Based on Chromosome 1 or Chromosome 2 (0.25 MB DOC) [file ppat.1000178.s004.doc]

# Figure S4 : AGC Clusters Based on Chromosome 1 or Chromosome 2

Legend : The Bp strains were clustered using the accessory genes on only either (A) Chromosome 1 or (B) Chromosome 2. In both the Chr 1 and Chr 2 clusters, a large set of clade C strains are discernible (red circles), and the strains in this clade are identical to the Clade C strains in Figure 3 in the Main Text. For both the Chr 1 and Chr 2 diagrams, a distinct set of clade A (blue circle) and clade E (green circle) strains are also discernible. Strains in clades A and E are identical for both Chr 2 and Figure 3 in the Main Text. For the Chr 1 diagram, one C clade branch now behaves as an outlier (green rectangle), and 4 strains formerly from the A clade in Figure 3 now segregate in the E clade (blue rectangle).

**Chr 1 AGC Clustering**

**A)**

**B)**

**Chr 2 AGC Clustering**
