# Supplementary material for: The Core and Accessory Genomes of Burkholderia pseudomallei: Implications for Human Melioidosis
Source: PLoS Pathog. 2008 Oct 17;4(10):e1000178. doi: 10.1371/journal.ppat.1000178 (PMC2564834; doi:10.1371/journal.ppat.1000178)
Supplement: Table S1 — Bp Isolates Used in this Study (0.17 MB DOC) [file ppat.1000178.s005.doc]

**Table S1: Bp Isolates Used in this Study**

| Isolate Category (No.) | Isolate ID | Source | Geography | Yr | ST* | MLST Allele at the following locus:* |
| --- | --- | --- | --- | --- | --- | --- |
|  |  |  |  |  |  | ace-gltB-gmhD-lepA-lipA-nark-ndh |
| Reference | K96243 | Human | Thailand | 1996 | - | - |
| Type strains (2) | ATCC23343 | Human | Thailand | 1996 | 51 | 3-1-2-3-1-4-3 |
|  | ATCC15682 | Monkey | Singapore | 1996 | 51 | 3-1-2-3-1-4-3 |
| Clinical (52) | 9-D38465 | Human Blood | Universiti Malaya, Malaysia | 1997 | - | - |
|  | 0-D10468 | Human Blood | Universiti Malaya, Malaysia | 1997 | 84 | 3-1-11-4-5-4-6 |
|  | 9-A57203 | Human Blood | Universiti Malaya, Malaysia | 1997 | 46 | 3-1-2-1-1-3-3 |
|  | 3-D85239 | Human Blood | Universiti Malaya, Malaysia | 1997 | - | - |
|  | 358 | Human Non septicemia | Universiti Malaya, Malaysia | 1997 | 84 | 3-1-11-4-5-4-6 |
|  | 4-D82316 | Human Non septicemia | Universiti Malaya, Malaysia | 1997 | 414 | 3-1-2-1-8-4-3 |
|  | 65676 | Human Non septicemia | Universiti Malaya, Malaysia | 1997 | 169 | 1-1-2-3-8-4-3 |
|  | 10 | Human Blood-Dead | NUS, Singapore | 1996 | 289 | 3-4-11-4-5-4-6 |
|  | 11 | Human Blood-Dead | NUS, Singapore | 1996 | 84 | 3-1-11-4-5-4-6 |
|  | 56 | Human Blood | NUS, Singapore | 2000 | 422 | 3-1-3-1-8-4-3 |
|  | 20 | Human Pus-Dead | NUS, Singapore | 1996 | - | - |
|  | 35 | Human Pus-dead | NUS, Singapore | 1996 | - | - |
|  | 4 | Human Pus-alive | NUS, Singapore | 1996 | - | - |
|  | 17 | Human Pus | NUS, Singapore | 2000 | 423 | 3-4-11-4-1-4-6 |
|  | 48 | Human Pus | NUS, Singapore | 2000 | 423 | 3-4-11-4-1-4-6 |
|  | 33 | Human Sputum-Alive | NUS, Singapore | 1996 | 423 | 3-4-11-4-1-4-6 |
|  | 8 | Human Sputum-Dead | NUS, Singapore | 1996 | 84 | 3-1-11-4-5-4-6 |
|  | 22 | Human Sputum-Dead | NUS, Singapore | 1996 | - | - |
|  | 6 | Human Sputum | NUS, Singapore | 2000 | - | - |
|  | 54 | Human Pericardial fluid | NUS, Singapore | 2000 | 423 | 3-4-11-4-1-4-6 |
|  | 59 | Human Urine-dead | NUS, Singapore | 1996 | 51 | 3-1-2-3-1-4-3 |
|  | KAS | Human | NUS, Singapore | 1997 | 422 | 3-1-3-1-8-4-3 |
|  | JAM | Human | NUS, Singapore | 1997 | - | - |
|  | 4090-0390 | Human | NUH, Singapore | 2004 | - | - |
|  | 4094-0081 | Human | NUH, Singapore | 2004 | 423 | 3-4-11-4-1-4-6 |
|  | D7 3230-3018 | Human | NUH, Singapore | 2002 | 423 | 3-4-11-4-1-4-6 |
|  | I6 4043-3096 | Human | NUH, Singapore | 2002 | - | - |
|  | D10 7310-3154 | Human | NUH, Singapore | 2002 | - | - |
|  | K1127 7244-293 | Human | NUH, Singapore | 2002 | - | - |
|  | DB30729/00 | Human | SGH, Singapore | 2002 | - | - |
|  | DB61901/00 | Human | SGH, Singapore | 2002 | - | - |
|  | DR08726/01 | Human | SGH, Singapore | 2002 | - | - |
|  | EM10266/01 | Human | SGH, Singapore | 2002 | 422 | 3-1-3-1-8-4-3 |
|  | DB19897/02 | Human | SGH, Singapore | 2002 | - | - |
|  | AH3 | Human | AH, Singapore | 2004 | - | - |
|  | AH4 | Human | AH, Singapore | 2004 | 51 | 3-1-2-3-1-4-3 |
|  | EB5661 | Human | CGH, Singapore | 2004 | 422 | 3-1-3-1-8-4-3 |
|  | EM1157 | Human | CGH, Singapore | 2004 | 51 | 3-1-2-3-1-4-3 |
|  | EM2107 | Human | CGH, Singapore | 2004 | - | - |
|  | 4009-19 | Human | TTH, Singapore | 2004 | - | - |
|  | 4094-225 | Human | TTH, Singapore | 2004 | 422 | 3-1-3-1-8-4-3 |
|  | 2635A | Human | ST70 isolate, Thailand | 2005 | - | - |
|  | 2693A | Human | ST70 isolate, Thailand | 2005 | - | - |
|  | 2694A | Human | ST70 isolate, Thailand | 2005 | - | - |
|  | 2663A | Human | ST70 isolate, Thailand | 2005 | - | - |
|  | 2747A | Human | ST70 isolate, Thailand | 2005 | - | - |
|  | 2664B | Human | ST70 isolate, Thailand | 2005 | - | - |
|  | 2667A | Human | ST70 isolate, Thailand | 2005 | - | - |
|  | 2614A | Human | ST70 isolate, Thailand | 2005 | - | - |
|  | 2689B | Human | ST70 isolate, Thailand | 2005 | - | - |
|  | 2644A | Human | ST70 isolate, Thailand | 2005 | - | - |
|  | 2684A | Human | ST70 isolate, Thailand | 2005 | - | - |
| Animal (19) | 363679 | Guinea pig | Universiti Malaya, Malaysia | 1997 | - | - |
|  | 216408 | Monkey | Universiti Malaya, Malaysia | 1997 | 51 | 3-1-2-3-1-4-3 |
|  | 490 | Bird of Paradise | NUS, Singapore | 1996 | 51 | 3-1-2-3-1-4-3 |
|  | P6 | Pig | NUS, Singapore | 1996 | 54 | 3-1-3-3-1-2-1 |
|  | 153 | Mueller gibbon | NUS, Singapore | 1996 | 51 | 3-1-2-3-1-4-3 |
|  | 107 | Doug Langur | NUS, Singapore | 1996 | - | - |
|  | 115 | Chimpanzee | NUS, Singapore | 1996 | 51 | 3-1-2-3-1-4-3 |
|  | 488 | Gorilla | NUS, Singapore | 1996 | 51 | 3-1-2-3-1-4-3 |
|  | 561 | Kangaroo | NUS, Singapore | 1996 | 51 | 3-1-2-3-1-4-3 |
|  | 612 | Crown Pigeon | NUS, Singapore | 1996 | 51 | 3-1-2-3-1-4-3 |
|  | G. Shepherd | German Shepherd | NUS, Singapore | 2000 | - | - |
|  | 10/96 | Pig | AVA, Singapore | 1997 | - | - |
|  | 15/96 | Pig | AVA, Singapore | 1997 | 51 | 3-1-2-3-1-4-3 |
|  | 21/96 | Pig | AVA, Singapore | 1997 | 46 | 3-1-2-1-1-3-3 |
|  | 27/96 | Pig | AVA, Singapore | 1997 | - | - |
|  | 35/96 | Pig | AVA, Singapore | 1997 | 51 | 3-1-2-3-1-4-3 |
|  | 457/96 | Pig | AVA, Singapore | 1997 | 51 | 3-1-2-3-1-4-3 |
|  | 497/96 | Pig | AVA, Singapore | 1997 | 46 | 3-1-2-1-1-3-3 |
|  | 504/96 | Pig | AVA, Singapore | 1997 | - | - |
| Environmental (20) | Soil | Field | Singapore | 1996 | 51 | 3-1-2-3-1-4-3 |
|  | 77/96 | Field | Singapore | 1996 | 51 | 3-1-2-3-1-4-3 |
|  | 79/96 | Field | Singapore | 1996 | 51 | 3-1-2-3-1-4-3 |
|  | 109/96 | Field | Singapore | 1996 | 51 | 3-1-2-3-1-4-3 |
|  | 12-40 | Pulau Tekong | Singapore | 2000 | - | - |
|  | 15-10 | Pulau Tekong | Singapore | 2000 | 423 | 3-4-11-4-1-4-6 |
|  | 15-10a | Pulau Tekong | Singapore | 2000 | 423 | 3-4-11-4-1-4-6 |
|  | 15-10b | Pulau Tekong | Singapore | 2000 | 423 | 3-4-11-4-1-4-6 |
|  | 15-10d | Pulau Tekong | Singapore | 2000 | - | - |
|  | 15-40 | Pulau Tekong | Singapore | 2000 | - | - |
|  | SW1T3 | Pulau Tekong | Singapore | 2000 | - | - |
|  | SW1T8 | Pulau Tekong | Singapore | 2000 | - | - |
|  | SW9T2 | Pulau Tekong | Singapore | 2000 | - | - |
|  | DB | Pulau Tekong | Singapore | 2000 | - | - |
|  | DC | Pulau Tekong | Singapore | 2000 | 51 | 3-1-2-3-1-4-3 |
|  | TRF661 | Soil | Singapore | 2000 | - | - |
|  | E38 | Soil | ST70 isolate, Thailand | 1994 | - | - |
|  | E358 | Soil | ST70 isolate, Thailand | 2002 | - | - |
|  | E359 | Soil | ST70 isolate, Thailand | 2002 | - | - |
|  | E371 | Soil | ST70 isolate, Thailand | 2003 | - | - |

* MLST sequence types. See Main Text for details
