# Supplementary material for: The Core and Accessory Genomes of Burkholderia pseudomallei: Implications for Human Melioidosis
Source: PLoS Pathog. 2008 Oct 17;4(10):e1000178. doi: 10.1371/journal.ppat.1000178 (PMC2564834; doi:10.1371/journal.ppat.1000178)
Supplement: Table S2 — Sequence identities between Bp K96243 and five Bp strains (S13, BP 1710a, 1710b, 1655, Pasteur) (0.03 MB DOC) [file ppat.1000178.s006.doc]

**Table S2: Sequence identities between Bp K96243 and five Bp strains (S13, BP 1710a, 1710b, 1655, Pasteur)**

(A) including GI genes and (B) excluding GI genes, on basis of the full genome and the 750 Bp-variable genes. The average sequence identities of the variable probes are significantly lower than the average sequence identities for the full genome in all five Bp strains, both in the presence and absence of previously known GI genes.

(A)

|  | **Including Genes in Known GIs** | | |
| --- | --- | --- | --- |
|  | **% identity** | | |
| **Bp Strain** | **All Genes** | **1-750** | **p-value** |
| K96243 | 100.00 | 100.00 |  |
| 1655 | 88.01 | 74.10 | 6.39E-19 |
| 1710a | 89.32 | 69.26 | 6.82E-34 |
| 1710b | 90.88 | 70.65 | 4.42E-34 |
| Pasteur | 90.10 | 73.57 | 1.21E-26 |
| S13 | 89.73 | 70.81 | 1.47E-31 |

(B)

|  | **Excluding Genes in Known GIs** | | |
| --- | --- | --- | --- |
|  | **% identity** | | |
| **Bp Strain** | **All Genes** | **1-750** | **p-value** |
| K96243 | 100.00 | 100.00 |  |
| 1655 | 90.56 | 87.39 | 8.49E-3 |
| 1710a | 93.56 | 91.00 | 6.38E-3 |
| 1710b | 95.19 | 92.80 | 6.93E-3 |
| Pasteur | 93.40 | 89.90 | 6.30E-4 |
| S13 | 93.19 | 86.72 | 2.46E-07 |
