# Supplementary material for: The Core and Accessory Genomes of Burkholderia pseudomallei: Implications for Human Melioidosis
Source: PLoS Pathog. 2008 Oct 17;4(10):e1000178. doi: 10.1371/journal.ppat.1000178 (PMC2564834; doi:10.1371/journal.ppat.1000178)
Supplement: Table S3 — Analysis of conserved metagenes* in the set of variable genes and non-variable genes in the B. pseudomallei genome, in the presence and absence of the GI genes (0.04 MB DOC) [file ppat.1000178.s007.doc]

**Table S3: Analysis of conserved metagenes* in the set of variable genes and non-variable genes in the *B. pseudomallei* genome, in the presence and absence of the GI genes.**

|  | **With GI Genes** | | **Without GI Genes** | |
| --- | --- | --- | --- | --- |
|  | **Variable** | **Core** | **Variable** | **Core** |
| Number of Metagenes | 217 | 2214 | 217 | 2214 |
| Number of Genes | 750 | 4619 | 519 | 4494 |
| Proportion | 0.29 | 0.48 | 0.42 | 0.49 |
| p-value | 8.68E-11 | | 2.9E-02 | |

* Metagenes are conserved between *B. pseduomallei*, *B. thailandenesis*, *B. mallei* and *B. cepacia*.
