# Supplementary material for: The Core and Accessory Genomes of Burkholderia pseudomallei: Implications for Human Melioidosis
Source: PLoS Pathog. 2008 Oct 17;4(10):e1000178. doi: 10.1371/journal.ppat.1000178 (PMC2564834; doi:10.1371/journal.ppat.1000178)
Supplement: Table S4 — Genes Present in Strains Associated with the AGC Clinical Clade (0.24 MB DOC) [file ppat.1000178.s008.doc]

**Table S4 : Genes Present in Strains Associated with the AGC Clinical Clade**

| **Gene ID** | **Chr** | **GI or Indel** | **Annotation** |
| --- | --- | --- | --- |
| BPSL0031 | 1 | . | flagellar biosynthetic protein |
| BPSL0113 | 1 | . | conserved hypothetical protein |
| BPSL0113A | 1 | . | putative exported protein |
| BPSL0129 | 1 | GI2 | prophage integrase |
| BPSL0130 | 1 | GI2 | conserved hypothetical phage protein |
| BPSL0130a | 1 | GI2 | hypothetical phage protein |
| BPSL0135 | 1 | GI2 | conserved hypothetical phage protein |
| BPSL0137 | 1 | GI2 | hypothetical phage protein |
| BPSL0141 | 1 | GI2 | putative phage DNA-binding protein |
| BPSL0142 | 1 | GI2 | putative phage-encoded membrane protein |
| BPSL0143 | 1 | GI2 | hypothetical phage protein |
| BPSL0144 | 1 | GI2 | putative phage protein |
| BPSL0145 | 1 | GI2 | putative phage protein |
| BPSL0146 | 1 | GI2 | putative phage-encoded membrane protein |
| BPSL0150 | 1 | GI2 | phage major tail sheath protein |
| BPSL0151 | 1 | GI2 | putative phage tail fiber assembly protein |
| BPSL0152 | 1 | GI2 | phage-related tail fiber protein |
| BPSL0153 | 1 | GI2 | putative phage protein |
| BPSL0155 | 1 | GI2 | phage baseplate assembly protein |
| BPSL0156 | 1 | GI2 | phage baseplate assembly protein |
| BPSL0157 | 1 | GI2 | phage-encoded modification methylase |
| BPSL0160 | 1 | GI2 | phage tail completion protein |
| BPSL0162 | 1 | GI2 | putative phage-encoded lipoprotein |
| BPSL0163 | 1 | GI2 | putative phage-encoded peptidoglycan binding protein |
| BPSL0164 | 1 | GI2 | putative phage-encoded membrane protein |
| BPSL0168 | 1 | GI2 | phage head completion/stabilization protein |
| BPSL0169 | 1 | GI2 | phage terminase, endonuclease subunit |
| BPSL0170 | 1 | GI2 | phage major capsid protein precursor |
| BPSL0171 | 1 | GI2 | putative phage capsid scaffolding protein |
| BPSL0172 | 1 | GI2 | phage terminase, ATPase subunit |
| BPSL0176 | 1 | GI2 | putative phage-encoded membrane protein |
| BPSL0550 | 1 | GI3 | hypothetical protein |
| BPSL0553 | 1 | GI3 | putative DNA-binding protein |
| BPSL0554 | 1 | GI3 | hypothetical phage protein |
| BPSL0555 | 1 | GI3 | putative membrane protein |
| BPSL0559 | 1 | GI3 | hypothetical protein |
| BPSL0563 | 1 | GI3 | hypothetical protein |
| BPSL0564 | 1 | GI3 | hypothetical protein |
| BPSL0565 | 1 | GI3 | hypothetical protein |
| BPSL0566 | 1 | GI3 | hypothetical protein |
| BPSL0568 | 1 | GI3 | hypothetical protein |
| BPSL0569 | 1 | GI3 | conserved hypothetical protein |
| BPSL0570 | 1 | GI3 | conserved hypothetical protein |
| BPSL0571 | 1 | GI3 | putative membrane protein |
| BPSL0572 | 1 | GI3 | hypothetical protein |
| BPSL0573 | 1 | GI3 | putative exported protein |
| BPSL0574 | 1 | GI3 | subtilase family protein |
| BPSL0575 | 1 | GI3 | hypothetical protein |
| BPSL0577 | 1 | GI3 | phage integrase family protein |
| BPSL0583 | 1 | GI3 | hypothetical protein |
| BPSL0585 | 1 | GI3 | hypothetical protein |
| BPSL0587 | 1 | GI3 | phage integrase family protein |
| BPSL0588 | 1 | GI3 | hypothetical protein |
| BPSL0736 | 1 | nGI1 | hypothetical protein |
| BPSL0737 | 1 | nGI1 | hypothetical protein |
| BPSL0738 | 1 | nGI1 | hypothetical protein |
| BPSL0739 | 1 | nGI1 | hypothetical protein |
| BPSL0741 | 1 | nGI2 | conserved hypothetical protein |
| BPSL0742 | 1 | nGI2 | putative membrane protein |
| BPSL0743 | 1 | nGI2 | conserved hypothetical protein |
| BPSL0744 | 1 | nGI2 | putative phage-related integrase |
| BPSL0745 | 1 | GI4 | hypothetical protein |
| BPSL0746 | 1 | GI4 | hypothetical protein |
| BPSL0748 | 1 | GI4 | hypothetical protein |
| BPSL0750 | 1 | GI4 | hypothetical protein |
| BPSL0752 | 1 | GI4 | hypothetical protein |
| BPSL0753 | 1 | GI4 | hypothetical protein |
| BPSL0754 | 1 | GI4 | putative integrase/recombinase (fragment) |
| BPSL0758 | 1 | GI4 | putative phosphoesterase |
| BPSL0759 | 1 | GI4 | hypothetical protein |
| BPSL0761 | 1 | GI4 | hypothetical protein |
| BPSL0763 | 1 | GI4 | putative helicase SNF2 family protein |
| BPSL0764 | 1 | GI4 | hypothetical protein |
| BPSL0765 | 1 | GI4 | putative helicase family protein |
| BPSL0766 | 1 | GI4 | hypothetical protein |
| BPSL0767 | 1 | GI4 | putative phospholipase protein |
| BPSL0768 | 1 | GI4 | conserved hypothetical protein |
| BPSL0769 | 1 | GI4 | hypothetical protein |
| BPSL0770 | 1 | GI4 | conserved hypothetical protein |
| BPSL0772 | 1 | GI4 | phage integrase family protein |
| BPSL0939 | 1 | . | putative DeoR family regulatory protein |
| BPSL0941 | 1 | . | hypothetical protein |
| BPSL0943 | 1 | . | putative insertion element protein |
| BPSL0944 | 1 | GI5 | putative phage integrase/recombinase protein |
| BPSL0945 | 1 | GI5 | conserved hypothetical protein |
| BPSL0946 | 1 | GI5 | conserved hypothetical protein |
| BPSL0947 | 1 | GI5 | putative type I restriction enzyme specificity protein |
| BPSL0948 | 1 | GI5 | putative type I restriction-modification methylase |
| BPSL0949 | 1 | GI5 | hypothetical protein |
| BPSL0952 | 1 | GI5 | putative replication protein |
| BPSL1092 | 1 | . | putative membrane protein |
| BPSL1112 | 1 | . | putative lipoprotein |
| BPSL1137 | 1 | GI6 | hypothetical protein |
| BPSL1138 | 1 | GI6 | hypothetical protein |
| BPSL1140 | 1 | GI6 | putative phage-related protein |
| BPSL1142 | 1 | GI6 | putative phage-related protein |
| BPSL1143 | 1 | GI6 | putative phage terminase |
| BPSL1144 | 1 | GI6 | putative exported protein |
| BPSL1151 | 1 | GI6 | hypothetical protein |
| BPSL1154 | 1 | GI6 | hypothetical protein |
| BPSL1157 | 1 | GI6 | putative phage integrase |
| BPSL1406A | 1 | . | hypothetical protein |
| BPSL1639 | 1 | GI8 | putative transposase (fragment) |
| BPSL1642 | 1 | GI8 | putative GntR-family regulatory protein |
| BPSL1646 | 1 | GI8 | putative monooxygenase |
| BPSL1648 | 1 | GI8 | conserved hypothetical protein |
| BPSL1650 | 1 | GI8 | putative ABC transport system, permease protein |
| BPSL1651 | 1 | GI8 | putative ABC transport system, permease protein |
| BPSL1652 | 1 | GI8 | putative ABC transport system, ATP-binding protein |
| BPSL1654 | 1 | GI8 | succinate-semialdehyde dehydrogenase [NADP+] |
| BPSL1655 | 1 | GI8 | putative outer membrane porin protein |
| BPSL1656 | 1 | GI8 | hypothetical protein |
| BPSL1694 | 1 | GI8 | putative transposase (fragment) |
| BPSL1696 | 1 | GI8 | putative recombinase |
| BPSL1702 | 1 | GI8 | putative invertase |
| BPSL1703 | 1 | GI8 | transposase (fragment) |
| BPSL1704 | 1 | GI8 | transposase |
| BPSL1704a | 1 | GI8 | hypothetical protein |
| BPSL1705 | 1 | GI8 | putative membrane protein |
| BPSL1707 | 1 | GI8 | putative exported oxidase |
| BPSL1708 | 1 | GI8 | putative exported protein |
| BPSL1708A | 1 | GI8 | putative insertion element (fragment) |
| BPSL1709 | 1 | GI8 | putative non-ribosomal peptide synthase (fragment) |
| BPSL2039 | 1 | . | putative membrane protein |
| BPSL2087A | 1 | . | hypothetical protein |
| BPSL2088 | 1 | . | hypothetical protein |
| BPSL2332 | 1 | . | hypothetical protein |
| BPSL2574 | 1 | GI9 | hypothetical protein |
| BPSL2579 | 1 | GI9 | hypothetical protein |
| BPSL2580 | 1 | GI9 | hypothetical protein |
| BPSL2581 | 1 | GI9 | hypothetical protein |
| BPSL2583 | 1 | GI9 | hypothetical protein |
| BPSL2586 | 1 | GI9 | putative phage integrase |
| BPSL3113 | 1 | . | putative integrase (fragment) |
| BPSL3115 | 1 | GI10 | putative transcriptional regulator |
| BPSL3118 | 1 | GI10 | putative restriction modification system methylase |
| BPSL3257 | 1 | GI11 | putative plasmid recombinase |
| BPSL3259 | 1 | GI11 | putative plasmid conjugal transfer protein |
| BPSL3260 | 1 | GI11 | conserved hypothetical protein |
| BPSL3262 | 1 | GI11 | putative plasmid conjugal transfer protein |
| BPSL3263 | 1 | GI11 | putative plasmid conjugal transfer protein |
| BPSL3264 | 1 | GI11 | putative plasmid conjugal transfer protein |
| BPSL3267 | 1 | GI11 | hypothetical protein |
| BPSL3268 | 1 | GI11 | putative membrane protein |
| BPSL3269 | 1 | GI11 | hypothetical protein |
| BPSL3270 | 1 | GI11 | putative plasmid replication protein |
| BPSL3343 | 1 | GI12 | putative bacteriophage protein |
| BPSL3344 | 1 | GI12 | putative bacteriophage integrase |
| BPSL3345 | 1 | GI12 | putative bacteriophage-related protein |
| BPSL3346 | 1 | GI12 | hypothetical protein |
| BPSL3347 | 1 | GI12 | putative bacteriophage-related lipoprotein |
| BPSL3348 | 1 | GI12 | putative bacteriophage protein |
| BPSL3349 | 1 | GI12 | putative membrane protein |
| BPSL3351 | 1 | GI12 | putative bacteriophage coat protein |
| BPSS0068 | 2 | . | hypothetical protein |
| BPSS0380A | 2 | GI13 | hypothetical protein |
| BPSS0383 | 2 | GI13 | putative DNA-binding protein |
| BPSS0384 | 2 | GI13 | hypothetical protein |
| BPSS0384A | 2 | GI13 | hypothetical protein |
| BPSS0385 | 2 | GI13 | hypothetical protein |
| BPSS0386 | 2 | GI13 | transposon Tn2501 resolvase |
| BPSS0387 | 2 | GI13 | putative phage-related protein |
| BPSS0388 | 2 | GI13 | putative phage-related protein |
| BPSS0389 | 2 | GI13 | putative phage-related protein |
| BPSS0391 | 2 | GI13 | putative phage-related hypothetical protein |
| BPSS0391A | 2 | GI13 | hypothetical protein |
| BPSS0400 | 2 | . | putative bacteriophage protein |
| BPSS0632 | 2 | . | patatin-like phospholipase |
| BPSS0732 | 2 | . | hypothetical protein |
| BPSS1048c | 2 | GI15 | hypothetical bacteriophage protein |
| BPSS1049 | 2 | GI15 | hypothetical bacteriophage protein |
| BPSS1050 | 2 | GI15 | hypothetical bacteriophage protein |
| BPSS1052 | 2 | GI15 | hypothetical bacteriophage replication protein |
| BPSS1053 | 2 | GI15 | hypothetical bacteriophage-acquired protein |
| BPSS1055 | 2 | GI15 | putative partition protein |
| BPSS1057 | 2 | GI15 | putative bacteriophage gp29 protein |
| BPSS1058 | 2 | GI15 | putative bacteriophage gp30 protein |
| BPSS1059 | 2 | GI15 | putative bacteriophage gp31 protein |
| BPSS1060 | 2 | GI15 | hypothetical bacteriophage protein |
| BPSS1062 | 2 | GI15 | putative phage protein |
| BPSS1063 | 2 | GI15 | putative bacteriophage terminase, ATPase subunit |
| BPSS1065 | 2 | GI15 | putative major capsid protein precursor |
| BPSS1066 | 2 | GI15 | putative bacteriophage terminase, endonuclease subunit |
| BPSS1067 | 2 | GI15 | putative bacteriophage head completion/stabilization protein |
| BPSS1072 | 2 | GI15 | putative bacteriophage-acquired protein |
| BPSS1073 | 2 | GI15 | putative bacteriophage protein |
| BPSS1074 | 2 | GI15 | putative bacteriophage tail completion protein R |
| BPSS1076 | 2 | GI15 | hypothetical bacteriophage protein |
| BPSS1077 | 2 | GI15 | putative site-specific DNA methyltransferase |
| BPSS1078 | 2 | GI15 | putative bacteriophage baseplate assembly protein V |
| BPSS1082 | 2 | GI15 | putative bacteriophage protein gp17 |
| BPSS1083 | 2 | GI15 | putative bacteriophage-acquired protein |
| BPSS1084 | 2 | GI15 | putative bacteriophage major tail sheath protein |
| BPSS1087 | 2 | GI15 | putative bacteriophage membrane protein |
| BPSS1088 | 2 | GI15 | putative bacteriophage tail-related protein |
| BPSS1089 | 2 | GI15 | putative bacteriophage late control gene D protein |
| BPSS1385 | 2 | . | putative ATP/GTP binding protein |
| BPSS2047 | 2 | . | putative chrolohydrolase |
| BPSS2048 | 2 | . | putative glutathione S-transferase |
| BPSS2058 | 2 | GI16 | putative ATP-binding inner membrane transport protein |
| BPSS2059 | 2 | GI16 | conserved hypothetical protein |
| BPSS2060 | 2 | GI16 | L-asparaginase |
| BPSS2061 | 2 | GI16 | conserved hypothetical protein |
| BPSS2061A | 2 | GI16 | putative transposase (fragment) |
| BPSS2062 | 2 | GI16 | acetyltransferase (GNAT) family protein |
| BPSS2063 | 2 | GI16 | hypothetical protein |
| BPSS2064 | 2 | GI16 | putative porin protein |
| BPSS2065 | 2 | GI16 | putative fatty aldehyde dehydrogenase |
| BPSS2067 | 2 | GI16 | putative aldose 1-epimerase |
| BPSS2068 | 2 | GI16 | short chain dehydrogenase |
| BPSS2069 | 2 | GI16 | ABC transporter, ATP-binding protein |
| BPSS2070 | 2 | GI16 | branched-chain amino acid transport system permease |
| BPSS2071 | 2 | GI16 | putative exported protein |
| BPSS2072 | 2 | GI16 | mandelate racemase / muconate lactonizing enzyme |
| BPSS2074 | 2 | GI16 | senescence marker protein-30 (SMP-30) family protein |
| BPSS2075 | 2 | GI16 | conserved hypothetical protein |
| BPSS2076 | 2 | GI16 | transposase IS66 family protein (pseudogene) |
