# Supplementary material for: The Core and Accessory Genomes of Burkholderia pseudomallei: Implications for Human Melioidosis
Source: PLoS Pathog. 2008 Oct 17;4(10):e1000178. doi: 10.1371/journal.ppat.1000178 (PMC2564834; doi:10.1371/journal.ppat.1000178)
Supplement: Table S5 — Primer pairs for amplification of housekeeping loci in multilocus sequence typing analysis (MLST) (0.04 MB DOC) [file ppat.1000178.s009.doc]

**Table S5: Primer pairs for amplification of housekeeping loci in multilocus sequence typing analysis (MLST).**

| Locus | Primers | Forward (5’🡪 3’) | Reverse (5’🡪 3’) |
| --- | --- | --- | --- |
| ace | Outer-ace | GCCGCTCGGCGCTTCTCAAA | AGCCGCCGTTCAGCGAAAAATC |
| gltB | Outer-gltB | TGTCGCGGCCCGTCTTCATCT | ATCAGCACCGAGGCGCATACGAC |
| gmhD | Outer-gmhD | TCGCGCAGGGCACGCAGTT | GGCTGCCGACCGTGAGACC |
| lepA | Outer-lepA | GCATCACGACGCCGACGTAGTTGT | CTGGCGGGCCTTTTCGGCTAAA |
| lipA | Outer-lipA | ACGATCCGACCGCCAAGCAGAAGG | ACGTACTCGCGCACCGGCAGATGG |
| narK | Outer-narK | GCCGTCAGCGTGAGCCTCGTCT | AGCCCGCGTTCTGCAACCACA |
| Ndh | Outer-ndh | TATCCCGCCGATCAGAAACAGTCC | GCCCGGCTCGCCCTCGTC |

**Primers used for validation tests.**

| Type of  validation | Primer | Forward (5’🡪3’) | Reverse (5’🡪3’) |
| --- | --- | --- | --- |
| Validation of variable probes | BPSL2707 | gtctccgaccacacgatgat | tgagcagattaccgacagga |
|  | BPSL0137 | cgagggactgttgcttgag | attcaccgacgagatgacg |
|  | BPSL0900 | ggctggtattgggtcgtc | cgagcgtgagcagatagaac |
|  | BPSS0681 | ctgtcgttctttcgctgctc | gggatgtcggctggagtat |
|  | BPSS2076 | acaggcgtatcgtcagcat | cattgggcatctggaagac |
|  | BPSS2295 | gaagtggctgtccgatgac | cgctggatgaagttgaagaag |
| Validation of novel GIs | BPSL2363 | catccacttcttcagcacga | cgtcaaaaccgcattcaag |
|  | BPSL2364 | gtggtttctgctcgtctgc | ctccttgaccttcggcatt |
|  | BPSL2365 | atgaacctgccaacacctct | acaaagccagtccgtcag |
|  | BPSL2701 | tttttccgtactggctcgac | cgcgacgatagttgacgac |
|  | BPSL2702 | gagttcggcatcgtcatagc | catggcttcggtatctcctc |
|  | BPSL2703 | ctgaaggtgagcgacgagtt | atttgttccgttcgttgtcg |
