# Supplementary material for: The Core and Accessory Genomes of Burkholderia pseudomallei: Implications for Human Melioidosis
Source: PLoS Pathog. 2008 Oct 17;4(10):e1000178. doi: 10.1371/journal.ppat.1000178 (PMC2564834; doi:10.1371/journal.ppat.1000178)
Supplement: Text S1 — Supplementary Methods (0.04 MB DOC) [file ppat.1000178.s010.doc]

**Text S1 : Supplementary Methods**

**Identification of Cross-Species Homologs (Metagenes)**

We identified metagenes across four related bacterial species of the *Burkholderia* genus: *B. cenocepacia* (*Bc*), *B. mallei* (*Bm*), *B. pseudomallei* (*Bp*) and *B. thailandensis* (*Bt*). Genome sequences for *B. pseudomallei* (*Bp*) K96243, *B. mallei* (*Bm*) ATCC 23344 and *B. thailandensis* (*Bt*) E264 were obtained from Genbank under accession numbers (Chr 1 / Chr 2) *Bp* (BX571965/BX571966) [1], *Bm* (CP000010 / CP000011) [2], and *Bt* (NC_007651 / NC_007650) [3]. The *B. cenocepacia* (*Bc*) J2315 genome was downloaded from the *Burkholderia Cenocepacia* Sequencing website (ftp://ftp.sanger.ac.uk/pub/bc).

Using the *Bp* genome as a reference, 3460 Chr 1 and 2395 Chr 2 open reading frames (ORFs) were queried against the *Bc*, *Bm* and *Bt* genomes using tblastn [4]. To minimize the number of ambiguous predictions including ORFs with matches to multiple genomic locations, we constrained the resulting matches to have I) a minimum length of 50 amino acids (aa), II) a minimal e-value cut-off of 1e-6 and III) a minimum percent identity of 50%. *Bc* Chr 3 was excluded from analysis as only 27 metagenes (<1%) were gained from its inclusion. Homology assignments returned 2675 genes and were validated by a reciprocal blast assay resulting in 2590 genes. Control analyses using either *Bc*, *Bm* or *Bt* as starting reference genomes yielded similar metagene sets (data not shown). A more comprehensive analysis of the metagenes can be found in Ref. 5

**References**:

1. Holden, M. T., Titball, R. W., Peacock, S. J., Cerdeno-Tarraga, A. M., Atkins, T., et al. (2004) Genomic plasticity of the causative agent of melioidosis, Burkholderia pseudomallei. Proc Natl Acad Sci U S A 101**:** 14240-5.

2. Nierman, W. C., DeShazer, D., Kim, H. S., Tettelin, H., Nelson, K. E., et al. (2004) Structural flexibility in the Burkholderia mallei genome. Proc Natl Acad Sci U S A 101**:** 14246-51.

3. Yu, Y., Kim, H. S., Chua, H. H., Lin, C. H., Sim, S. H., et al. (2006) Genomic patterns of pathogen evolution revealed by comparison of Burkholderia pseudomallei, the causative agent of melioidosis, to avirulent Burkholderia thailandensis. BMC Microbiol 6**:** 46.

4. Altschul, S. F., Gish, W., Miller, W., Myers, E. W. & Lipman, D. J. (1990) Basic local alignment search tool. J Mol Biol 215**:** 403-10.

5. Lin CH, Bourque G, Tan P (2008). A Comparative Synteny Map of *Burkholderia* Species Links Large-scale Genome Rearrangements to Fine-scale Nucleotide Variation in Prokaryotes. Mol. Biol. Evol. 25:549-558
