# Supplementary material for: The Core and Accessory Genomes of Burkholderia pseudomallei: Implications for Human Melioidosis
Source: PLoS Pathog. 2008 Oct 17;4(10):e1000178. doi: 10.1371/journal.ppat.1000178 (PMC2564834; doi:10.1371/journal.ppat.1000178)
Supplement: Text S2 — Expression Patterns of GI Genes During Bp Growth (0.57 MB DOC) [file ppat.1000178.s011.doc]

# Text S2 : Expression Patterns of GI Genes During Bp Growth

Using Bp microarrays, we have previously demonstrated that close to 20% of all Bp genes are transcriptionally regulated during growth in rich media (Rodrigues et al., 2006). These “growth-regulated” genes could be grouped into several independent expression clusters, each exhibiting distinctive temporal patterns and specific associations with particular growth phases and transition points (eg early phase to log phase, or log to stationary phase). For example, a large cluster of 185 genes, containing genes essential for general biosynthesis (eg protein translation, nucleotide transport and metabolism) exhibited a sharply defined pattern of high expression at early phase with a rapid shut-off upon entry into log phase (Rodrigues et al., 2006). Genes in the same expression cluster tended to share similar cellular functions, as has also been reported for other micro-organisms (Laub et al., 2000; Bozdech et al., 2003). Furthermore, many of these genes were often expressed just before those functions were required by the microbe. For example, we observed upregulated expression of several quorum sensing genes just prior to the population initiating exponential growth, which would coincide with a period of rapid expansion where population numbers would need to be tightly monitored. Taken collectively, these results are consistent with Bp employing a ‘just-in-time’ manufacturing strategy where genes are expressed only when their associated proteins are required.

In the current report, we surmised that if any GI gene also displayed a similarly complex pattern of gene expression during growth, that such GI genes might play a role in Bp growth and biology. We thus revisited the gene expression microarray data set from Rodrigues et al., 2006, and found several clear examples of GI genes exhibiting growth-associated gene expression. As shown in the Figure, during early phase there was upregulated expression of one GI2 gene (*BPSL0141*), two GI3 genes (*BPSL0549* and *BPSL0563*), and one GI 8 gene (*BPSL1704*). In contrast, during the early to log transition, genes on GI4, GI6 and GI16 were upregulated. Finally, upon entry into stationary phase, we observed concordant upregulation of multiple genes on GIs 3 and 13(see Figure). The striking expression patterns of these GI genes during Bp growth suggests that the transcriptional circuitry regulating gene expression in the core genome during growth may have adapted to also regulate these GI genes, thus raising the possibility that a subset of GI genes may have functional roles related to Bp growth.

Legend : Expression patterns of GI genes during Bp growth. Genes on the GIs were queried against a microarray data set of Bp growth-regulated gene expression (Rodrigues et al., 2006). The top bar represents the three major phases of Bp growth (early, log, and stationary). Genes on the various GIs are indicated. The scale bar at the bottom indicates the range of gene expression variation.

## References

Bozdech, Z., M. Llinas, B. L. Pulliam, E. D. Wong, J. Zhu, and J. L. DeRisi. 2003. The transcriptome of the intraerythrocytic developmental cycle of *Plasmodium falciparum*. PLoS Biol 1:E5.

Laub, M. T., H. H. McAdams, T. Feldblyum, C. M. Fraser, and L. Shapiro. 2000. Global Analysis of the Genetic Network Controlling a Bacterial Cell Cycle. Science 290:2144-2148.

Rodrigues F, Sarkar-Tyson M, Harding SV, Sim SH, Chua HH, et al. (2006) Global map of growth-regulated gene expression in *Burkholderia* *pseudomallei*, the causative agent of melioidosis. J Bacteriol 188: 8178-8188.
